# Maternal SARS-CoV-2 impacts fetal placental macrophage programs and placenta-derived microglial models of neurodevelopment

**DOI:** 10.1186/s12974-024-03157-w

**Published:** 2024-06-25

**Authors:** Lydia L. Shook, Rebecca E. Batorsky, Rose M. De Guzman, Liam T. McCrea, Sara M. Brigida, Joy E. Horng, Steven D. Sheridan, Olha Kholod, Aidan M. Cook, Jonathan Z. Li, Donna K. Slonim, Brittany A. Goods, Roy H. Perlis, Andrea G. Edlow

**Affiliations:** 1 Vincent Center for Reproductive Biology, Massachusetts General Hospital, 55 Fruit Street, Thier Research Building, 903B, Boston, MA 02114 USA; 2 Department of Obstetrics, Gynecology and Reproductive Biology, Harvard Medical School, Boston, MA USA; 3 Data Intensive Studies Center, Tufts University, Boston, MA USA; 4 Center for Genomic Medicine, Massachusetts General Hospital, Boston, MA USA; 5 Department of Psychiatry, Massachusetts General Hospital, Harvard Medical School, Boston, MA USA; 6 Thayer School of Engineering and Program, Dartmouth College, Hanover, NH USA; 7 Department of Molecular and Systems Biology, Geisel School of Medicine, Dartmouth College, Lebanon, NH USA; 8 Department of Medicine, Brigham and Women’s Hospital, Harvard Medical School, Boston, MA USA; 9 Department of Computer Science, Tufts University, Medford, MA USA

**Keywords:** Hofbauer cells, Microglia, Single-cell RNA sequencing, Fetal brain, Placenta, Neurodevelopment, Neuroimmune, SARS-CoV-2, COVID-19

## Abstract

**Background:**

The SARS-CoV-2 virus activates maternal and placental immune responses. Such activation in the setting of other infections during pregnancy is known to impact fetal brain development. The effects of maternal immune activation on neurodevelopment are mediated at least in part by fetal brain microglia. However, microglia are inaccessible for direct analysis, and there are no validated non-invasive surrogate models to evaluate *in utero* microglial priming and function. We have previously demonstrated shared transcriptional programs between microglia and Hofbauer cells (HBCs, or fetal placental macrophages) in mouse models.

**Methods and results:**

We assessed the impact of maternal SARS-CoV-2 on HBCs isolated from 24 term placentas (*N* = 10 SARS-CoV-2 positive cases, 14 negative controls). Using single-cell RNA-sequencing, we demonstrated that HBC subpopulations exhibit distinct cellular programs, with specific subpopulations differentially impacted by SARS-CoV-2. Assessment of differentially expressed genes implied impaired phagocytosis, a key function of both HBCs and microglia, in some subclusters. Leveraging previously validated models of microglial synaptic pruning, we showed that HBCs isolated from placentas of SARS-CoV-2 positive pregnancies can be transdifferentiated into microglia-like cells (HBC-iMGs), with impaired synaptic pruning behavior compared to HBC models from negative controls.

**Conclusion:**

These findings suggest that HBCs isolated at birth can be used to create personalized cellular models of offspring microglial programming.

**Supplementary Information:**

The online version contains supplementary material available at 10.1186/s12974-024-03157-w.

## Background

Multiple population-based studies have suggested that maternal infection during pregnancy may have a transgenerational impact on offspring neurodevelopment. Initial work found that the incidence of schizophrenia was increased after influenza pandemics in Finland [1], Denmark [2], and the UK [3]. Subsequent registry studies directly examining the association of maternal infection requiring hospitalization during pregnancy with diagnoses of autism and other neurodevelopmental disorders in offspring also found risk to be increased [4, 5]. Using electronic health records, we identified an increased risk of delayed acquisition of speech and motor milestones, beyond that attributable to prematurity, in a US cohort of offspring whose mothers had SARS-CoV-2 during pregnancy [6, 7]. Similarly, authors of a prospective cohort study of 127 children in Brazil found an increased risk of neurodevelopmental delay with *in utero* exposure to maternal SARS-CoV-2 infection [8], and a recent meta-analysis of smaller studies identified additional evidence of neurodevelopmental sequelae – including reductions in fine motor and problem-solving skills – in infants with *in utero* SARS-CoV-2 exposure compared to unexposed and pre-pandemic cohorts [9]. If these early signals foreshadow an increased risk of neurodevelopmental disorders in childhood and adulthood, the public health implications could be profound, given the significant number of pregnancies exposed to SARS-CoV-2 infection.

Despite the convergence of studies suggesting that maternal viral infection may increase offspring risk for neurodevelopmental disorders, the precise biological mechanisms leading to offspring neurodevelopmental vulnerability in SARS-CoV-2 are not known. Direct placental and fetal infection with SARS-CoV-2 virus is uncommon based on current evidence [10–14], and thus vertical transmission is unlikely to be a major cause of neurodevelopmental sequelae. Animal models of maternal immune activation (MIA), in which offspring of pregnant dams treated with an immune stimulus recapitulate the behavioral hallmarks of human neurodevelopmental disorders, have been used for decades to investigate candidate *in utero* mechanisms of neurodevelopmental programming [15–18]. Embryonic microglia have emerged as central mediators of offspring neuropathology in the setting of MIA [15]. However, microglia from surviving offspring are inaccessible for direct analysis in humans, necessitating alternative models for evaluating the impact of SARS-CoV-2 on the fetal brain.

Prior work from our group has identified remarkable similarities in the transcriptional programs and reactivity of fetal placental macrophages, or Hofbauer cells (HBCs), and fetal brain microglia isolated from mouse embryos [19, 20]. These two cell types share an embryonic origin in the fetal yolk sac [21, 22], and both carry the imprint of the *in utero* environment, with fetal yolk sac-derived macrophages serving as the progenitors for the lifelong pool of microglia [23, 24]. Here, we investigate the impact of SARS-CoV-2 exposure on the transcriptional profiles of HBC subpopulations to gain insight into fetal resident tissue macrophage programming. Our results demonstrate that HBCs are a heterogeneous cell type, with eight subpopulations exhibiting distinct cellular programs, and that maternal SARS-CoV-2 infection is associated with varying impact on function in these subpopulations. Assessment of differentially expressed genes implies impaired phagocytosis in specific subclusters, a key function of both HBCs and microglia; we confirm these effects using a previously validated assay of microglial synaptic pruning via synaptosome phagocytosis [25–27]. In aggregate, we demonstrate the application of HBC-based cellular models to gain non-invasive insight into the impact of *in utero* exposures on fetal brain development.

## Results

### Hofbauer cells are a heterogeneous population with subclusters demonstrating both M1- and M2-like transcriptional signatures

Placental chorionic villous tissues were collected from *N* = 24 birthing individuals: *N* = 10 individuals who had a positive SARS-CoV-2 nasopharyngeal PCR test during pregnancy, and *N* = 14 individuals with a negative PCR at delivery and no history of a positive SARS-CoV-2 test during pregnancy. In the majority of SARS-CoV-2 positive maternal cases, infections occurred remote from delivery (median [IQR]: 12.2 [0.8–19.6] weeks prior to delivery) in unvaccinated individuals. No placental samples were infected with SARS-CoV-2 at delivery (defined as having detectable SARS-CoV-2 viral load in a validated assay sensitive to 40 copies/mL) and individuals tested negative for SARS-CoV-2 by PCR at admission for delivery [14, 28]. For simplicity, we will use the nomenclature “SARS-CoV-2 positive” to refer to cases of maternal infection with SARS-CoV-2 during pregnancy and “SARS-CoV-2 negative” to refer to controls, i.e. no history of maternal infection with SARS-CoV-2 during pregnancy. Additional participant characteristics are provided in Table 1, results of placental pathology examination are provided in Supplementary File S8, and known offspring neurodevelopmental outcomes are detailed in Table S2. Offspring neurodevelopmental outcomes should be interpreted with the caveats that participant numbers are too small to draw conclusions about association or causality, and that fewer than half of children with neurodevelopmental disorders will have received a diagnosis by 5 years of age [29].

**Table 1:** Clinical information of study participants. GA: Gestational age. M: male. F: female. N/A: not applicable. COVID-19 infections occurred in unvaccinated individuals, with the exception of D18 who had completed the primary mRNA vaccine series 7 weeks prior to conception. ^1^COVID-19 severity was defined by NIH criteria (Available at https://www.covid19treatmentguidelines.nih.gov/overview/clinical-spectrum/). ^2^Maternal age is provided as a range to preserve participant anonymity. Control subjects D1 and D6 were diagnosed with a hypertensive disorder of pregnancy at delivery not requiring medical therapy; no other participants had a hypertensive disorder of pregnancy. Newborn 5-minute APGARs were > 7 for all participants. Additional clinical information is provided in Supplementary Data File S8

Study ID	Maternal SARS-CoV-2 infection in pregnancy	COVID-19 severity^1^	GA at SARS-CoV-2 infection (weeks)	Infant Sex	Maternal Age^2^ (years)	GA at Delivery (weeks)	Infant birthweight (grams)
D1	No	N/A	N/A	M	36–40	39.3	3200
D2	No	N/A	N/A	M	26–30	38.9	3450
D3	No	N/A	N/A	M	26–30	38.9	3350
D4	No	N/A	N/A	M	26–30	40.3	3670
D5	No	N/A	N/A	F	36–40	39	2785
D6	No	N/A	N/A	M	31–35	37.1	3360
D7	No	N/A	N/A	M	41–45	39	2850
D8	No	N/A	N/A	F	< 26	40.4	3270
D9	Yes	Mild	11.3	M	31–35	39.9	3370
D10	Yes	Mild	28.3	M	26–30	40.1	3125
D11	Yes	Severe	24.7	M	31–35	39	3395
D12	Yes	Mild	16.3	M	26–30	40.3	3985
D13	Yes	Mild	32.4	M	31–35	38.6	3660
D14	Yes	Asymptomatic	22.6	F	36–40	40.7	4260
D15	Yes	Mild	38.9	F	26–30	38.9	3365
D16	Yes	Asymptomatic	38	F	31–35	38.1	3340
D17	Yes	Severe	37.6	F	< 26	38.6	3135
D18	Yes	Mild	28	M	31–35	40.6	3495
D19	No	N/A	N/A	M	31–35	39.7	3385
D20	No	N/A	N/A	M	26–30	39.9	3350
D21	No	N/A	N/A	F	36–40	40.1	3330
D22	No	N/A	N/A	M	31–35	40.1	3530
D23	No	N/A	N/A	F	31–35	39.6	2850
D24	No	N/A	N/A	F	31–35	40.4	3350

To assess the HBC transcriptome, we first used a previously-described protocol to obtain primarily HBCs from placental villi; in this protocol, a Percoll-based gradient and negative bead-based selection steps are used to isolate putative HBCs from other cell types present in the chorionic villi (including trophoblasts, fibroblasts) [30]. Single-cell RNA sequencing was performed on cell suspensions from *N* = 4 SARS-CoV-2 positive cases with symptomatic COVID-19 disease and *N* = 8 SARS-CoV-2 negative controls (10x Genomics). Demographic and clinical information for these participants are presented in Table 1 (D1-12).

After quality control filtering to remove putative doublets and cells with less than 300 identified genes, we obtained a dataset comprised of a total of 70,817 cells. We then performed sample integration and graph-based clustering to identify broad cell types (Figure S1A). Based on analyses of marker gene expression (Figure S1B), we found that the majority of cells in our dataset had marker gene expression consistent with monocytes/macrophages, and that other cell types were represented to a lesser extent, including some fibroblasts, vascular endothelial cells (VECs), extravillous trophoblasts (EVTs), leukocytes (NK cells, CD8 + T cells, B cells), neutrophils and red blood cells (Figure S1C). From this dataset, we excluded all cell types that were not identifiable as macrophages/monocytes. After additional quality control filtering for nUMIs, gene counts, and percent mitochondrial reads (see Methods), this resulted in a dataset containing 31,719 high-quality placental macrophages/monocytes. All subsequent analyses were performed with this final dataset.

**Fig. 1:**
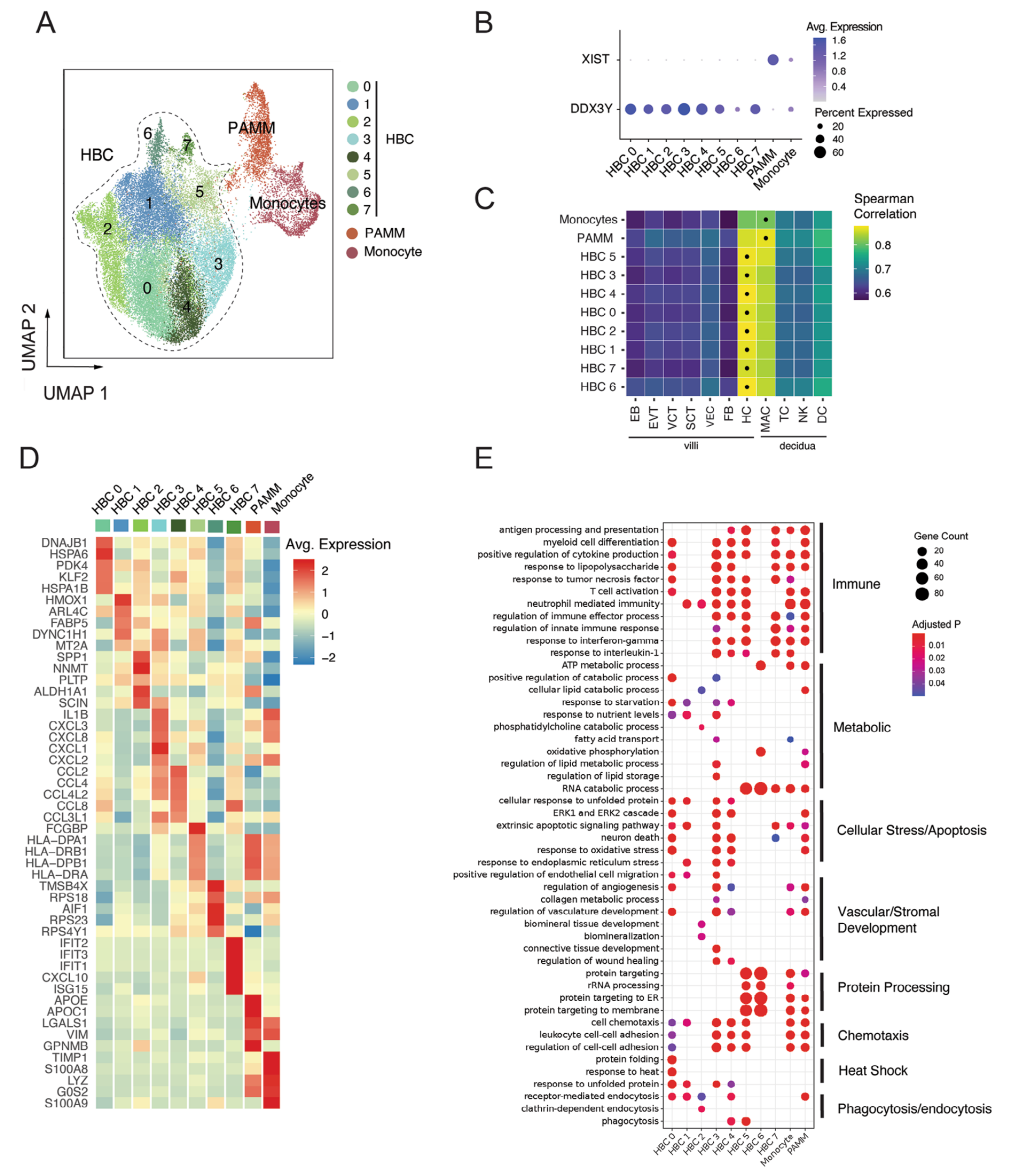
Transcriptomic profiles of fetal and maternal macrophages and monocytes isolated from term placentas with and without SARS-CoV-2 infection during pregnancy. (**A**) Uniform Manifold Approximation and Projection (UMAP) visualization of 31,719 high-quality placental macrophage and monocyte cells enriched from placentas of pregnancies with (*N* = 4) and without (*N* = 8) SARS-CoV-2 infection shows 10 clusters. HBC: Hofbauer cell; PAMM: placenta-associated maternal monocyte/macrophage. (**B**) Cluster-specific expression of *DDX3Y*, expressed only in fetal cells, and *XIST*, expressed only in maternal cells, in placentas from individuals carrying a male fetus (*N* = 10). (**C**) Correlation of cluster-average gene expression with annotated cell types identified by Suryawanshi et al., *Sci Adv*, 2018. Each heatmap shows Spearman correlation coefficients. Highest correlation coefficient per cluster is indicated by black dots. HBC clusters were most highly correlated with Suryawanshi HBC clusters, PAMM cluster most correlated with decidual macrophages. (**D**) Heatmap displaying expression (log_2_ fold change) of the top 5 marker genes per cluster. (**E**) Gene Ontology (GO) Biological Process enrichment analysis for cluster marker genes. GO terms displayed were curated from the top significant GO terms in each cluster, selecting the processes most relevant to macrophage function, and reducing redundancy. Gene Count gives the number of genes in the query set that are annotated by the relevant GO category. GO terms with an adjusted p-value < 0.05 were considered significantly enriched

After re-processing selected cells for quality control as described, we identified 10 total subclusters of macrophages/monocytes (Fig. 1A), with representation of each subcluster across donors from both SARS-CoV-2 positive cases and controls (Figure S2A). To distinguish HBCs, which are placental macrophages of fetal origin, from macrophages or monocytes of maternal origin, we used cells isolated from male placentas (*N* = 10). Male fetal origin was confirmed by high expression of *DDX3Y* and low expression of *XIST* in 8 subclusters; these were labeled HBC 0–7 (Fig. 1B). The macrophage cluster with high expression of *XIST* (consistent with maternal origin) was annotated as placenta-associated maternal macrophages and monocytes (PAMMs, Fig. 1B) [31]. A small cluster of monocytes – identified as such by high expression of monocyte marker genes *S100A8*, *S100A9*, and *TIMP1* – demonstrated equal expression levels of both *DDX3Y* and *XIST*, suggesting that both fetal and maternal cells were present in this monocyte cluster. To further support HBC cluster annotation, we next compared the overall gene expression profiles of each cluster to a previously published single-cell dataset derived from human first-trimester placenta and decidua [32]. In this analysis, all putative HBC subclusters showed highest correlation with HBC expression profiles from this dataset, whereas the monocyte and PAMM clusters had higher correlation with decidual macrophages than HBCs (Fig. 1C).

To delineate differences in the identity and functions of HBC subclusters, we next assessed top marker genes defining each subcluster, shown as an average heatmap (Fig. 1D); the complete list of marker genes by subcluster is available in Supporting Data Values File (S1). Marker genes for HBC clusters 3, 4, 5, and 7 suggested involvement in classic M1 macrophage/pro-inflammatory activities. HBC cluster 3 demonstrated high expression of chemokine (C-X-C motif) ligand genes (*CXCL*s) as well as pro-inflammatory marker genes *IL1B*, *IL1A*, *TNF*, and *NFKB1*. HBC cluster 4 demonstrated high expression of multiple CC chemokine ligand genes (CCLs) in a profile similar to that observed in HBCs responding to lipopolysaccharide stimulation in vitro [33]. HBC cluster 5 was characterized by high expression of genes encoding major histocompatibility complex (MHC) class II molecules (human leukocyte antigen (HLA)-DRA/B1 and -DP) and Fc-gamma binding protein (FCGBP), suggesting a role in antigen presentation to CD4 + T cells. MHC class II molecules are critical to antigen-specific responses, and upregulation of HLA complexes and antigen presentation pathways has been observed in proteomic analyses of HBCs stimulated with the viral dsRNA mimic poly(I: C) [33]. HBC cluster 7 demonstrated marker genes from the interferon-induced protein with tetratricopeptide repeats (*IFIT*) family (*IFIT2*, *IFIT3*), *CXCL10*, *ISG15*, and *MX1*, associated with a pro-inflammatory type 1 interferon antiviral response. To gain further insight into the biological processes reflected in each HBC subcluster, we performed Gene Ontology (GO) enrichment analyses of cluster marker genes (Fig. 1E). GO biological process enrichment results are available in Supporting Data Values File (S2). As would be expected given marker gene expression noted previously, pathways involved in M1-like immune/inflammatory responses were enriched in HBC 3, 4, 5, and 7, including “response to interleukin-1,” “response to interferon-gamma,” “response to tumor necrosis factor,” and “positive regulation of cytokine production.”

Gene signatures of HBC clusters 0, 1, and 2 reflected engagement in specific stress response processes, particularly response to inflammation and/or tissue damage, to ultimately support placental function. HBC 0 and HBC 1 were characterized by genes encoding heat shock proteins and other proteins involved in endoplasmic reticulum stress and the unfolded protein response, such as *HSPA6, HSPA1B, DNAJB1, HSP90B1*, *HSPA5* and *BAG3*. The unfolded protein response represents a homeostatic response to restore balance when endoplasmic reticulum stress is sensed and to modulate and/or resolve inflammation [34]. Additionally, HBC 0’s high expression of *PDK4* and *KLF2* may suggest involvement in attenuating oxidative stress responses and reducing pro-inflammatory cytokine production [35, 36], and HBC 1’s high expression of *FABP5* and *HMOX1* suggests engagement in anti-inflammatory responses against heme-induced toxicity and induction towards M2 polarization [37, 38]. GO enrichment analysis of these clusters similarly demonstrated enrichment of pathways such as “response to unfolded protein”, “response to heat”, “response to endoplasmic reticulum stress”, and pathways related to cellular stress response and apoptosis (e.g. “extrinsic apoptotic signaling pathway”, “response to oxidative stress” and “ERK1 and ERK2 cascade”).

GO enrichment analysis also suggested both HBC 0 and HBC 1 were engaged in homeostatic functions including “receptor-mediated endocytosis”, “regulation of angiogenesis” and “vascular development”, and nutrient-sensing functions such as “response to nutrient levels” and “response to starvation.” HBC 2 was characterized by high expression of the genes encoding secreted phosphoprotein 1 (*SPP1*) and Nicotinamide N -methyltransferase (*NNMT*), both associated with M2 (anti-inflammatory) macrophage polarization in the context of tumor-associated macrophages [39, 40]; SPP1, also known as osteopontin, is secreted by HBCs and plays an important role in endothelial biology and angiogenesis [41]. GO analysis of HBC cluster 2 also demonstrated enrichment in “receptor-mediated endocytosis” (involved in intracellular transport of macromolecules), as well as “cellular lipid catabolic process,” and processes associated with stromal tissue development.

HBC 6 was characterized by expression of genes involved in regulation of actin polymerization and cytoskeleton organization (*TMSB4X* and *AIF1*, which encodes the canonical microglial marker Iba1 [42]) and several ribosomal proteins including *RPS18, RPS23*, and *RPS4Y1*. GO enrichment analysis of this cluster demonstrates highly specific enrichment of protein processing pathways (e.g. “protein targeting” pathways, “cytoplasmic translation,” “translational initiation”) and pathways related to RNA catabolism, oxidative phosphorylation, and ATP metabolism. Marker genes of the maternal PAMM subcluster included *APOE*, *APOC1*, *VIM, LGALS1*, and *GPNMB* among others, an expression pattern consistent with previously reported maternal placental macrophage transcriptional profiles [43, 44]. High expression of *LGALS1/3* and *GPNMB* by the PAMM cluster suggests a role in inflammation regulation [45–47], which was echoed by GO analyses identifying enrichment in immune response and immunomodulatory pathways (e.g. “antigen processing and presentation, “positive regulation of cytokine production”, and “regulation of innate immune response”). In addition, GO enrichment analysis demonstrated PAMM were engaged in lipid metabolic processes and receptor-mediated endocytosis, consistent with their known involvement in lipid engulfment, and tissue repair/scar formation [41, 43].

### Maternal SARS-CoV-2 infection drives cluster-specific differences in immune signaling and metabolic pathways

**Fig. 2:**
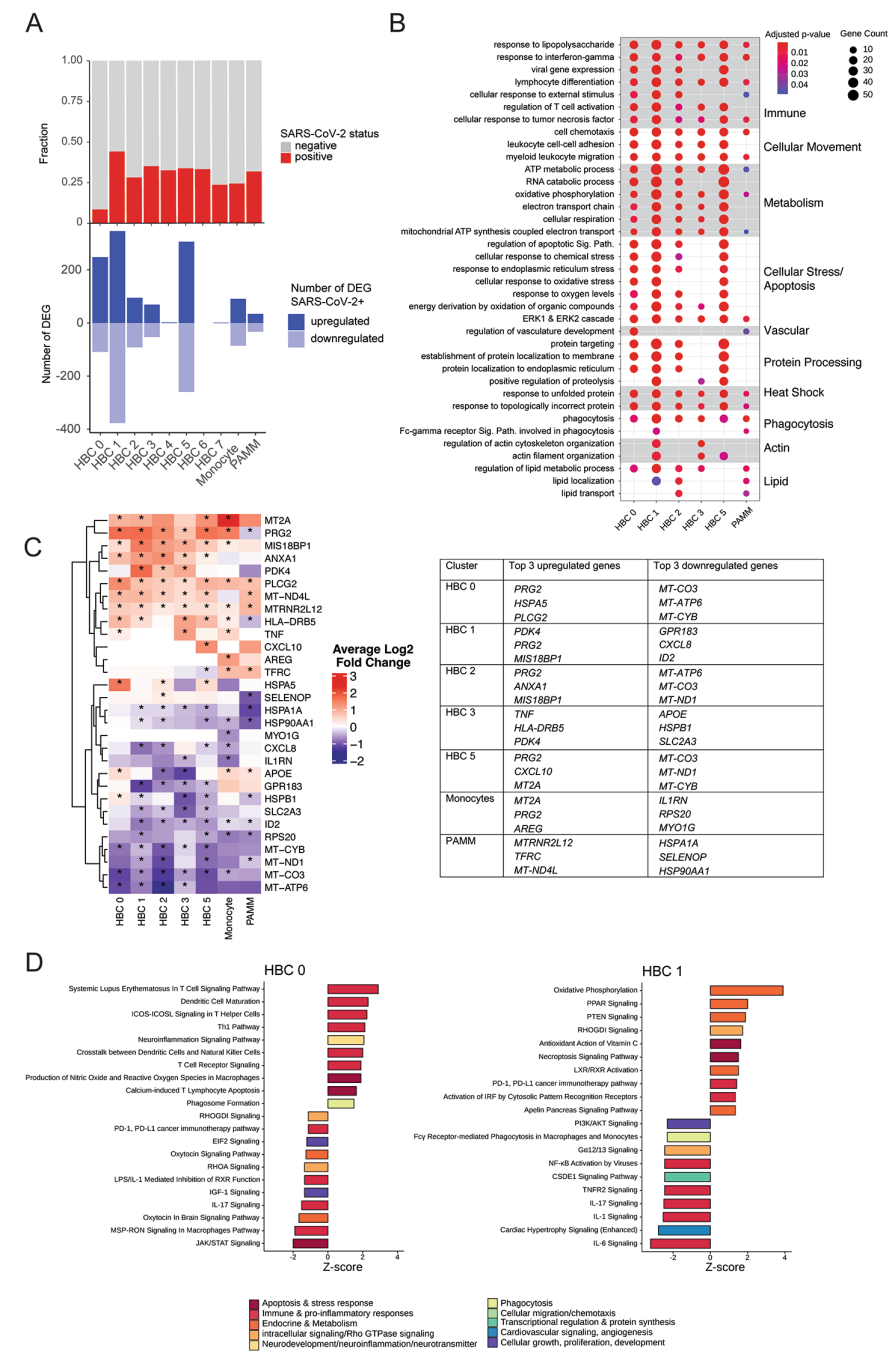
Impact of maternal SARS-CoV-2 infection on Hofbauer cell subclusters. DEG: differentially expressed genes. (**A**) Barplot demonstrating proportion of cells per cluster from SARS-CoV-2 positive cases (red) and negative controls (gray), top panel. Number of DEG upregulated (dark blue) and downregulated (light blue) by SARS-CoV-2 exposure per cluster, bottom panel. (**B**) Gene Ontology (GO) Biological Process enrichment analysis for DEG. Gene Count gives the number of genes in the query set that are annotated by the relevant GO category. GO terms with an adjusted p-value < 0.05 were considered significantly enriched. (**C**) Heatmap and table of the top 3 upregulated and downregulated DEG by SARS-CoV-2 by cluster. Color represents gene expression level (log_2_ fold change), *adjusted P-value < 0.05. (**D**) Ingenuity Pathway Analysis (IPA) of DEG for HBC clusters 0 (left) and 1 (right). Canonical pathways with absolute Z-score ≥ 1 and adjusted p-value < 0.05 are shown. IPA analysis for remaining HBC clusters depicted in Supplement

Once the baseline functions of HBC and PAMM subclusters had been established, we then sought to characterize the impact of maternal SARS-CoV-2 infection on the transcriptomic profile of HBC subclusters. To do so, we identified differentially expressed genes (DEG) by maternal SARS-CoV-2 status within each cluster. DEG were defined using log fold-change threshold of 0.2 and adjusted p-value of 0.05 (see Methods for full details). We first verified that each subcluster included representation from both SARS-CoV-2 + cases and controls (Fig. 2A, top panel). The proportion of cells from cases versus controls was consistent across all subclusters, except for HBC 0, which demonstrated a significantly greater contribution of control donor cells (Figure S2A). Of the 8 HBC clusters, a majority (5) were significantly impacted by maternal SARS-CoV-2 infection: HBC 0, 1, 2, 3, and 5 (Fig. 2A, bottom panel). In contrast, HBC clusters 4, 6, and 7 had very few DEG in the setting of maternal SARS-CoV-2 infection, with three, zero, and two DEG respectively (Supporting Data Values File S3). Of the 5 highly impacted clusters, HBC 1 and HBC 5 had the highest number of DEG by maternal SARS-CoV-2, with 723 and 566 DEG, respectively. PAMMs were impacted by SARS-CoV-2 to a lesser extent, with 67 DEG identified. Both up- and down-regulated DEG were identified across all impacted clusters. The complete list of DEG by SARS-CoV-2 infection status per subcluster are available in Supporting Data Values File (S3).

GO pathway enrichment analysis of DEG indicated that in the context of maternal SARS-CoV-2 infection, specific pathways involved in immune responses were enriched in all impacted HBC clusters (HBC 0, 1, 2, 3 and 5), including response to lipopolysaccharide, response to interferon-gamma, cellular response to tumor necrosis factor, and regulation of T cell activation (Fig. 2B, Supporting Data Values File S4). Additionally, all subclusters were enriched for pathways related to cellular movement, such as cell chemotaxis, leukocyte cell-cell adhesion, and myeloid leukocyte migration; heat shock-related pathways (unfolded protein response); and phagocytosis pathways.

GO enrichment analysis of DEG also indicated some biological processes that were only impacted in specific clusters in the setting of maternal SARS-CoV-2 infection (Fig. 2B-C). For example, regulation of vascular development was only implicated in HBC 0 and the PAMM cluster, and regulation of lipid metabolism and transport in all clusters except HBC 5. Cellular energy utilization pathways (e.g. ATP metabolism, electron transport chain/oxidative phosphorylation and cellular respiration), cellular stress/apoptosis pathways, and protein processing and actin cytoskeleton organization pathways were impacted in HBC clusters but not PAMMs. Taken together, these functional analyses suggest that in the context of maternal SARS-CoV-2 infection, HBC subclusters and PAMMs are differentially impacted, with key implicated biological processes including innate immune and pro-inflammatory signaling, cell chemotaxis and migration, cellular ATP and lipid metabolism, cellular phagocytosis, and the unfolded protein response.

To better understand the impact of SARS-CoV-2 on HBC functions, we next used Ingenuity Pathway Analysis (IPA), which predicts strength and directionality (i.e. activation or suppression) of enriched canonical pathways by subcluster. In this analysis, pathways with absolute Z-score value greater than 1 (consistent with IPA being able to predict a direction of dysregulation of the pathway) and Benjamini Hochberg-adjusted *p* < 0.05 were included and displayed by subcluster (Fig. 2C and Figure S2B-F, Supporting Data Values File S5). Z-scores ≥ 1 indicate upregulated signaling in the pathway and $\le$ 1 indicate downregulated pathway signaling [48]. Both HBC 1 and HBC 2 subclusters exhibited a primarily anti-inflammatory response to SARS-CoV-2, with activation of PPAR signaling and oxidative phosphorylation, a metabolic profile associated with an anti-inflammatory/pro-resolution phase macrophage signature [49, 50]. In HBC 1 and 2, suppression of IL-6, IL-1, and IL-17 pathways, and activation of LXR/RXR signaling pathways in SARS-CoV-2+ cases suggests involvement in resolution of inflammation, as LXR/RXR pathway activation in macrophages is associated with inhibition of inflammatory gene expression and promotion of lipid metabolism [51]. Also consistent with an anti-inflammatory role, HBC 2 showed strong suppression of the Coronavirus Pathogenesis Pathway and activation of Oxytocin Signaling Pathway, the latter of which is involved in attenuating oxidative and cellular inflammatory responses in macrophages [52].

Conversely, HBC 0 and 3 demonstrated primarily activated pro-inflammatory immune responses in SARS-CoV-2 positive cases, with increases in LPS/IL-1 mediated inhibition of RXR (HBC 3), Interferon induction (HBC 3), Neuroinflammation signaling (HBC 0 and 3), T-cell signaling (HBC 0 and 3), and Production of nitric oxide and reactive oxygen species (ROS) (HBC 0). Upregulation of apoptosis and ROS pathways in HBC cluster 0 may explain the significantly decreased representation of SARS-CoV-2 positive cases compared to controls in this cluster. Metabolic processes were suppressed in both clusters, including Oxytocin signaling pathway (HBC 0), Sirtuin signaling (HBC 3), and MSP-RON signaling (HBC 0 and 3) [52–54]. In the context of maternal SARS-CoV-2 infection, subcluster HBC 5 presented a mixed picture of both pro- and anti-inflammatory signaling, with upregulation of interferon, EIF 2, neuroinflammation and T cell related signaling pathways, balanced by upregulation of anti-inflammatory pathways such as PPAR signaling and downregulation of pro-inflammatory signaling pathways such as Coronavirus Pathogenesis pathway, FAK and TNF-mediated signaling pathways.

Compared to HBC subclusters, PAMMs were less impacted overall by maternal SARS-CoV-2 at a transcriptomic level, with 67 DEG identified. In the setting of maternal SARS-CoV-2 infection, PAMMs showed activation of pathways involved in immune responses including Production of Nitric Oxide and Reactive Oxygen Species, B-cell signaling pathways, Interferon induction, and activation of the pattern recognition receptor TREM-1 signaling. Similar pro-inflammatory patterns were observed for monocytes, including activation of antiviral response pathways and Th1 signaling pathways, and suppression of MSP-RON signaling (Figure S2C). Taken together, these analyses point to transcriptional shifts in some but not all subclusters in response to SARS-CoV-2, with a greater response by HBCs compared to PAMMs, driven by a combination of immune activation/pro-inflammatory signature in subclusters HBC 0 and HBC 3 and an anti-inflammatory tissue repair signature in clusters HBC 1 and HBC 2.

### Maternal SARS-CoV-2 infection impacts HBC transcriptional programs associated with phagocytosis, neuroinflammation, and neurological disorders

**Fig. 3:**
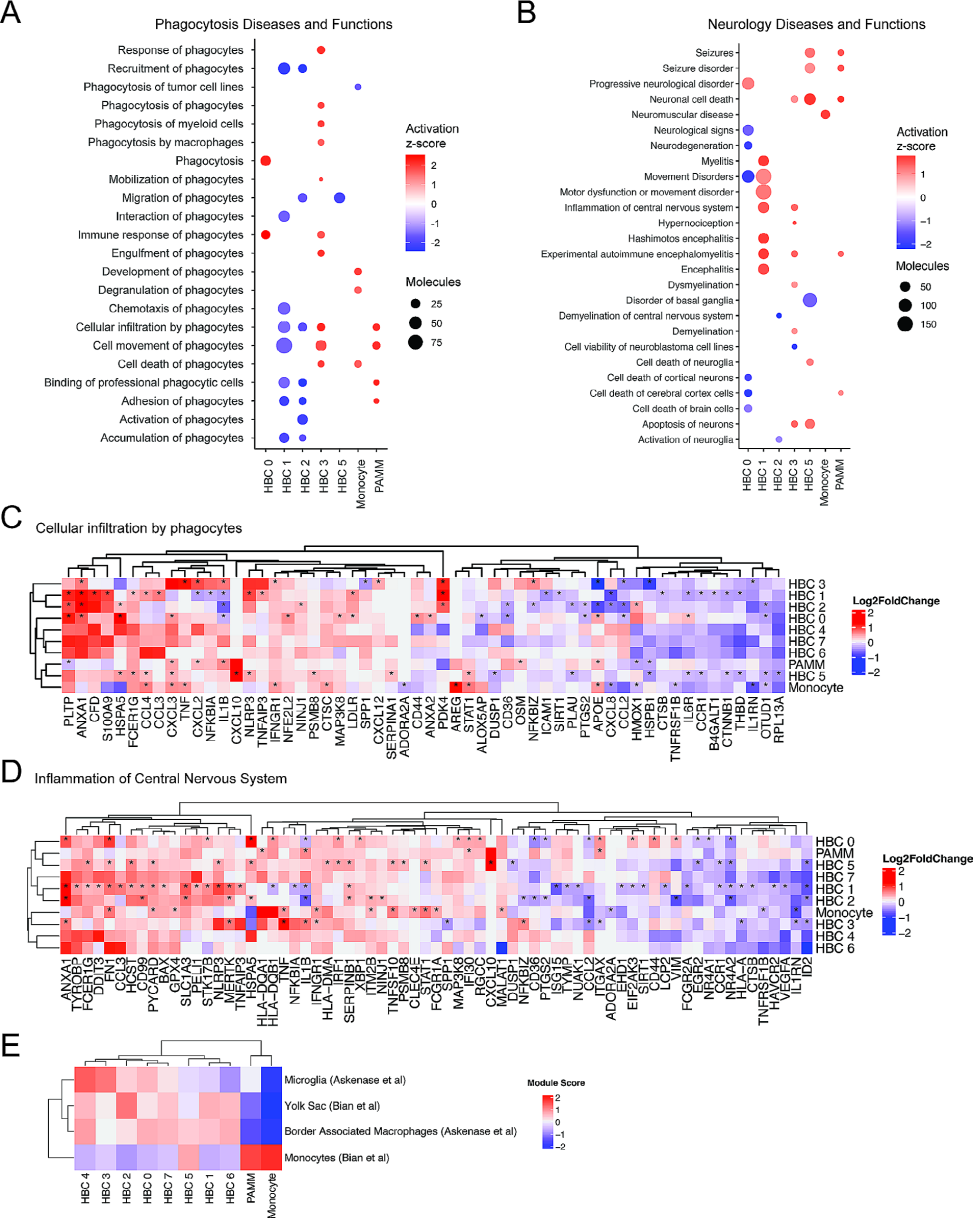
Impact of maternal SARS-CoV-2 on HBC gene programs associated with phagocytosis and neurologic disease. (**A-B**) Ingenuity Pathway Analysis (IPA) phagocytosis diseases and functions pathways (**A**), and neurologic diseases or functions (**B**), enriched for ≥ 3 DEGs, with absolute Z-score ≥ 1 and adjusted P-value < 0.05. Activation Z-score represented by color and number of DEGs by circle size, with red color indicating pathway activation and blue color indicating suppression. (**C-D**). Heatmap of gene expression in “Cellular Infiltration by Phagocytes” IPA Pathway (**C**) and “Inflammation of Central Nervous System” (**D**) by cluster. Color represents gene expression level (log_2_ fold change), *adjusted P-value < 0.05. (**E**). Module score by subcluster in comparison to cluster-specific gene expression of single-cell datasets from human brain: Microglia and Border Associated Macrophages (Askenase et al., *Sci Immunol*, 2021) and Yolk Sac Macrophages and Monocytes (Bian et al., *Nature*, 2020). Color indicates module score

Tissue-resident macrophages promote resolution of inflammation through phagocytosis of apoptotic cells, invading pathogens, or cellular debris [55, 56]. Phagocytosis is also a key function of microglia in early brain development [57–59]. IPA functional analysis of SARS-CoV-2-specific HBC signatures demonstrated that macrophage phagocytosis (Fig. 3A) and neurological disease-related pathways (Fig. 3B) were key functions and pathways implicated by the cluster-specific gene expression signatures (Supporting Data Values File S6). Figure 3 summarizes the impact of maternal SARS-CoV-2 infection on placental macrophage phagocytosis (Fig. 3A and C), illustrating the potential for altered HBC gene programs to provide insight into both fetal brain microglial function and the impact of maternal SARS-CoV-2 infection on neurodevelopment (Fig. 3B and D). These analyses predicted SARS-CoV-2-associated suppression of phagocyte chemotaxis and cell movement pathways (e.g. reduced “activation of phagocytes”, “recruitment of phagocytes”, “cell movement of phagocytes”, “adhesion of phagocytes”) in HBC 1, 2 and 5, consistent with the suppression of synaptosome phagocytosis (a proxy for synaptic pruning) observed in subsequent experiments using in vitro Hofbauer cell induced microglial assays, detailed below. In contrast to the consistent suppression of phagocytosis in HBC clusters 1, 2 and 5, HBC clusters 0 and 3 and the PAMM cluster demonstrated activation of phagocytosis-related pathways including “Phagocytosis” (HBC 0), “Immune response of phagocytes” (HBC 0), “Phagocytosis by macrophages” (HBC 3) and “Cellular infiltration by phagocytes” (HBC 3). A representative phagocytosis pathway from IPA and expression of its constituent genes by cluster is depicted as a heatmap in Fig. 3C. Cluster-specific alterations in phagocyte movement in the setting of maternal SARS-CoV-2 infection were primarily driven by expression differences in *CXCL2*, *NFKB1A*, *NFKB1Z*, *IL1B*, *CXCL8*, *CD36*, and *ICAM1* by cluster (Fig. 3C). Concordant with patterns observed in canonical pathways analyses, HBC 1 and 2 (and to a lesser extent HBC 5), which showed primarily immunomodulatory signatures, also show suppressed phagocytosis and phagocytic movement pathways, versus proinflammatory clusters HBC 0 and 3, which demonstrate activation of phagocytosis.

In addition to phagocytosis, pathways relevant to neurologic disease and microglial functions emerged as key dysregulated pathways in the setting of maternal SARS-CoV-2 infection. We therefore assessed whether DEG of HBC subclusters map to neuroinflammatory/neurodevelopmental pathways and functions in IPA, and plotted pathway activation Z-scores by subcluster (Fig. 3B). The transcriptional signature of HBC 1 – in which Fc-gamma receptor-mediated phagocytosis (Fig. 2C) and other previously-described phagocytosis pathways (Fig. 3A) are suppressed in SARS-CoV-2 + cases – is also associated with increased neuroinflammation, including positive activation Z-scores for “Inflammation of central nervous system”, “Myelitis”, and “Encephalitis.” Cluster-specific expression of the genes in the “Inflammation of central nervous system pathway” is depicted in Fig. 3D, with upregulation of signaling in this pathway driven by increased expression of *ANXA1*, *FN1*, *CCL3*, *SLC1A3, NLRP3*, and *MERTK*, among others. Interestingly, HBC 3 – in which “Inflammation of Central Nervous System” is also predicted to be activated after maternal SARS-CoV-2 infection and whose transcriptional signature is consistent with activation in phagocytosis pathways, also implicates increased “Apoptosis of neurons” and “Neuronal cell death” (Fig. 3B). In a developmental context, this pattern may represent a functional rather than pathologic gene signature in response to SARS-CoV-2, as microglia (resident brain macrophages) play a key role in neuronal cell turnover, regulation of neural progenitors, and synaptic rewiring in early neurodevelopment, all via phagocytosis [60]. Thus, increased phagocytosis by tissue-resident macrophages might be an adaptive response to SARS-CoV-2-associated inflammation, while reduced phagocytosis could be a pathologic or maladaptive response to maternal immune activation (e.g., reduced microglial phagocytosis and reduced synaptic pruning associated with maternal immune activation is thought to be a key aspect of the pathogenesis of autism spectrum disorder and other neurodevelopmental pathologies [61–63]). Taken together, these data support the concept that HBC transcriptional signatures provide insight into protective versus pathologic microglial programming in the setting of an immune challenge such as SARS-CoV-2.

Prior work from our group in a mouse model has shown that HBCs and fetal brain microglia share transcriptional profiles and responses to maternal obesity, an immune-activating exposure [20]. To further probe the potential connection between transcriptional signatures of HBC subclusters and brain microglia in humans, we next compared marker genes from HBC subclusters with gene modules from published human single cell atlases of macrophages derived from adult and embryonic brain (Fig. 3E, Supporting Data Values File S7) [64, 65]. Nearly all HBC subclusters scored highly for gene signatures found in microglia, yolk sac macrophages, or CNS border associated macrophages, compared with monocytes and PAMMs. HBC 2 and 1 exhibited greatest similarity to yolk sac macrophages whereas HBC 3 and 4 were most like microglia isolated from adult brain samples. In contrast, monocytes and PAMMs were most similar to circulating monocytes, which is concordant with their shared myeloid lineage [41]. This analysis supports the concept that HBCs isolated from full term human placenta share transcriptional signatures with yolk sac macrophages and fetal brain microglia, and thus may offer insights into global reprogramming of fetal macrophage populations, including those of the fetal brain, in the setting of maternal immune-activating exposures.

### HBCs isolated from placentas of SARS-CoV-2 positive pregnancies can be transdifferentiated to microglia-like cells (HBC-iMGs)

**Fig. 4:**
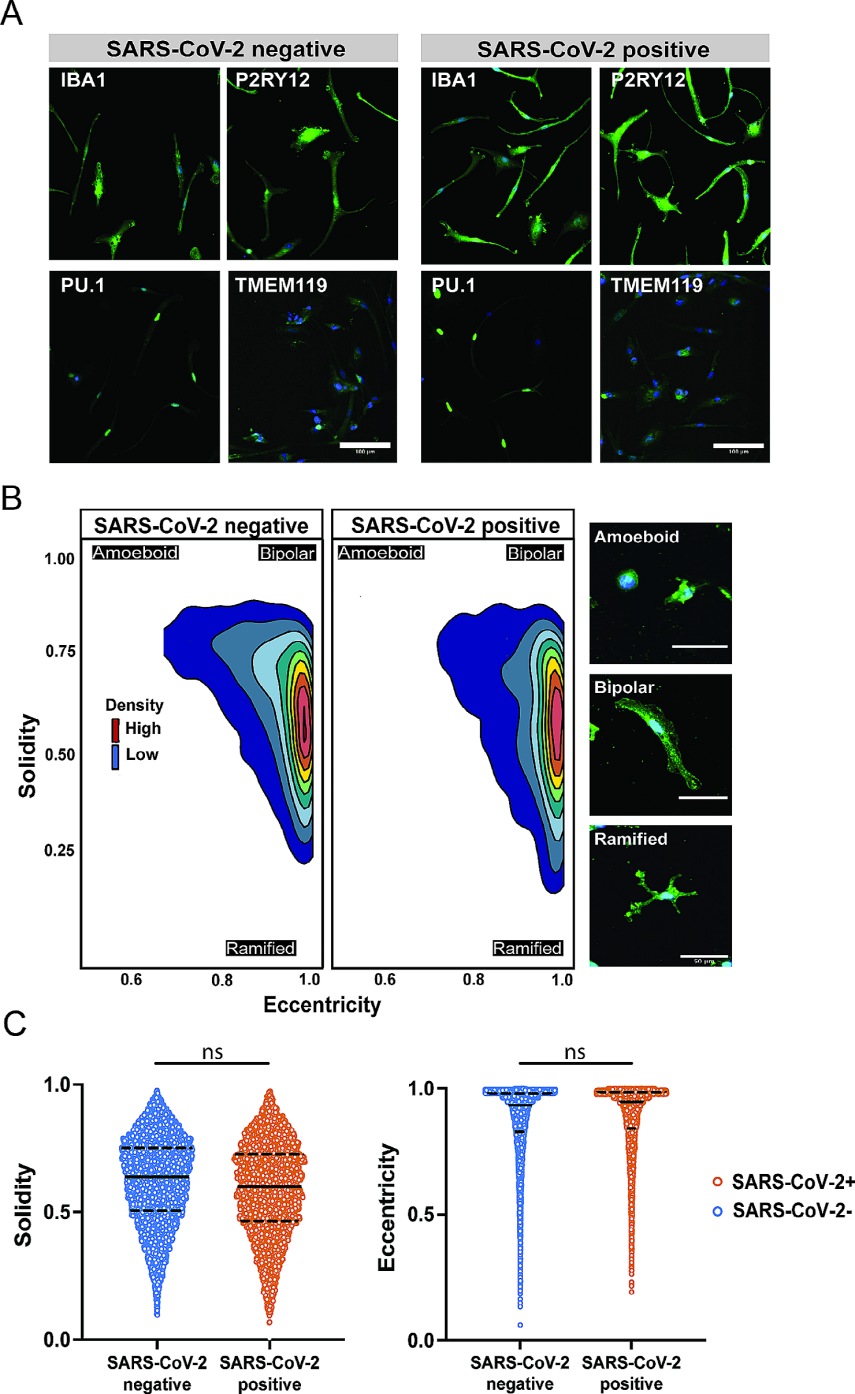
Phenotypic characterization of HBC-iMGs by direct cytokine reprogramming. HBC-iMGs: Hofbauer cells transdifferentiated toward microglia-like cells. (**A**) Representative images of HBC-iMGs, immunostained for microglial markers: IBA1, PU.1, P2RY12, TMEM119. Scale bar = 100 μm. (**B**) Morphology-smoothed density plots (solidity vs. eccentricity) for SARS-CoV-2 negative and positive samples as indicated. Representative confocal microscopy images of amoeboid, bipolar, and ramified HBC-iMGs. Scale bar = 50 μm. (**C**) Violin plots represent distribution of cell solidity (left) and eccentricity (right) measurements from SARS-CoV-2 negative controls (blue, *n* = 8965 cells from 9 participants) and positive cases (orange, *n* = 4754 cells from 10 participants). Solid lines represent median values and dashed lines interquartile range. Group differences assessed by linear mixed effects model. ns = not significant

To gain insight into the functional consequences of maternal SARS-CoV-2 infection on HBC populations, we used a model in which HBCs isolated from SARS-CoV-2 positive cases (*N* = 10) and SARS-CoV-2 negative controls (*N* = 9) were cultured in media containing IL-34 and GM-CSF to obtain transdifferentiated microglia-like cells (HBC-iMGs) (see Methods). Following culture, we assessed the expression of multiple markers associated with microglial identity, including IBA1, TMEM119, PU.1, P2RY12 and CX3CR1 [66, 67], and identified expression of all markers in the majority of HBC-iMGs from both SARS-CoV-2 positive cases and negative controls (Fig. 4A, Figure S3A-C).

### SARS-CoV-2-exposed HBC-iMGs demonstrate preserved morphology but impaired phagocytic behavior compared to HBC-iMGs from uninfected control placentas

To assess differences in cell phenotype by maternal SARS-CoV-2 infection, we first evaluated cellular morphology of IBA1-positive HBC-iMGs by quantitative assessment of two morphological characteristics: eccentricity (amoeboid vs. bipolar shape) and solidity (amoeboid/bipolar vs. ramified shape) (Fig. 4B-C), features extracted via segmentation using CellProfiler (Methods). More ramified microglial morphology in vivo is generally typical of resting-state, tissue-surveilling microglia in vivo [68, 69], while more amoeboid morphology is classically attributed to an immune-activated state [70], and typical of microglial patterns observed in fetal states [68, 71]. In this analysis, HBC-iMGs from SARS-CoV-2 negative controls demonstrated similar morphology to that observed in positive cases, reflected in solidity and eccentricity (Fig. 4B-C). In aggregate, our results suggest a lack of pronounced difference in microglial maturity or functional state, recognizing that no single measure captures activation state [72].

**Fig. 5:**
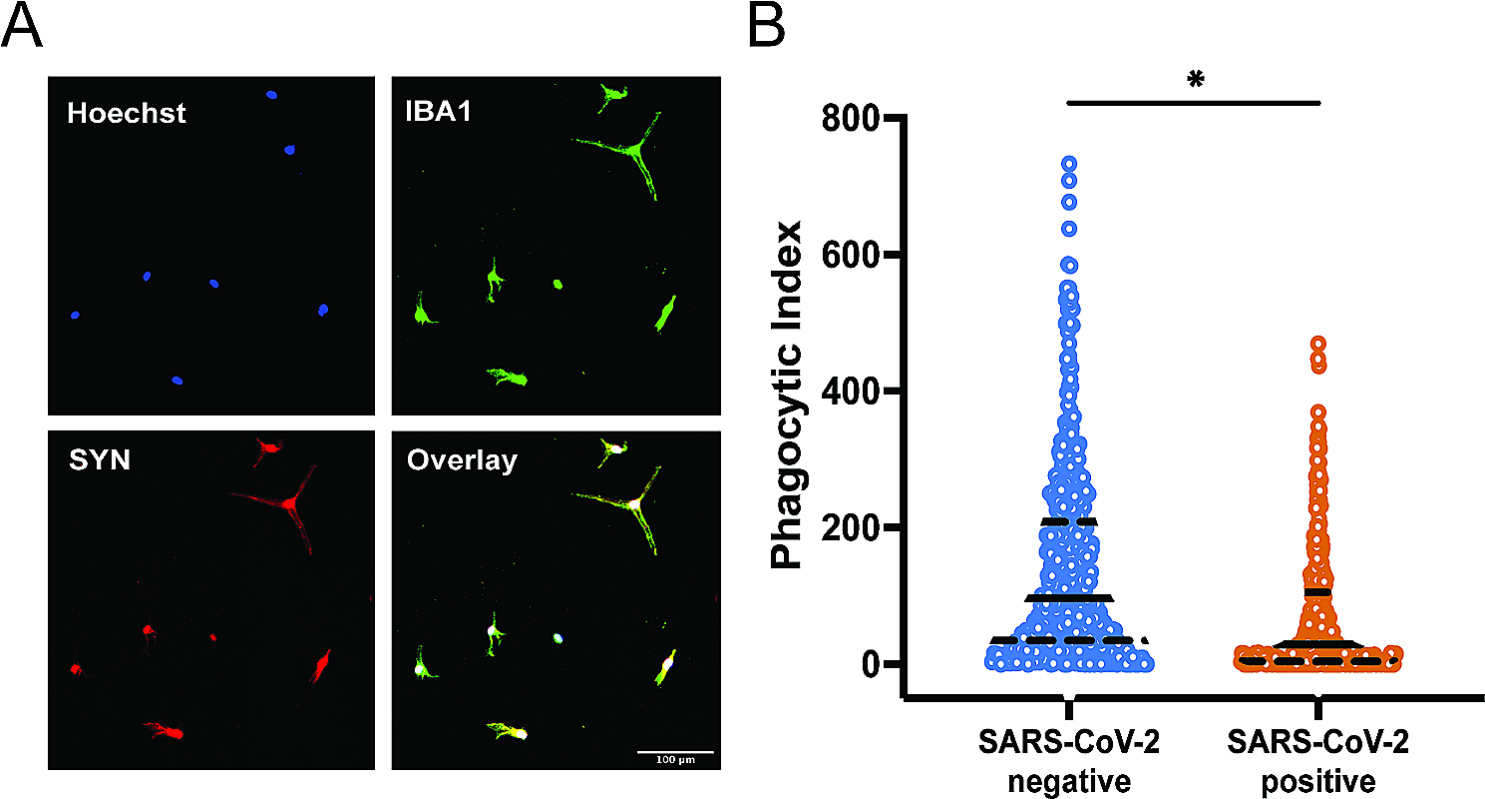
Impact of maternal SARS-CoV-2 on HBC-iMG synaptosome engulfment. HBC-iMGs: Hofbauer cells transdifferentiated toward microglia-like cells (**A**) Representative image showing colocalization of pHrodo-red labeled synaptosomes (SYN) and IBA1 positive HBC-iMGs. Hoechst = nuclear stain. Scale bar = 100 μm. (**B**) Violin plots of phagocytic index of image fields from phagocytosis assays of HBC-iMGs from 9 SARS-CoV-2 negative controls (blue, *n* = 484 fields) and 10 SARS-CoV-2 positive cases (orange, *n* = 394 fields). Phagocytic index is calculated as synaptosome area in pixels divided by cell count per image field. Solid lines represent median values and dashed lines interquartile range. Group differences assessed by linear mixed effects model. *P < 0.05

Transcriptional analyses of HBC clusters pointed to a cluster-specific impact of maternal SARS-CoV-2 on phagocytosis pathways. HBC clusters with the greatest similarity to embryonic/yolk sac microglia (e.g. HBC 1, 2) also exhibited cellular programs suggestive of impaired phagocytosis. Using a previously-validated model of synaptic pruning [25–27], a key physiologic function of microglia in early brain development, we next tested the functional capability of HBC-iMGs to engage in phagocytosis. In this assay, HBC-iMGs were co-cultured for 3 h with pHrodo Red-labeled neuronal synaptosomes derived from human induced pluripotent stem cells prior to fixation and imaging. This pH-sensitive label fluoresces following intracellular engulfment (see Methods). Synaptosome engulfment by IBA1-positive cells was then measured by quantifying fluorescence using confocal microscopy images with CellProfiler software applied for segmentation and thresholding (Fig. 5A). Compared to SARS-CoV-2 negative cases, HBC-iMGs from positive cases demonstrated significant impairment in synaptosome phagocytosis, reflected by a reduced mean phagocytic index and thus a decrease on average across all subpopulations of assayed cells (Fig. 5B). Phagocytic index was reduced across all SARS-CoV-2 positive samples, and was driven by reduced phagocytic uptake per cell, independent of the proportion of cells engaged in phagocytosis in any given sample. (Figure S3D). These functional analyses demonstrating reduced phagocytic capability following maternal SARS-CoV-2 infection are thus consistent with the transcriptomic signatures observed in a subset of HBC suggesting downregulation of phagocytosis.

## Discussion

Data from observational cohorts suggests an increased neurodevelopmental risk of offspring exposed *in utero* to maternal SARS-CoV-2 infection [6–8] but the underlying mechanism for offspring brain vulnerability remains unknown. Studies have consistently demonstrated that maternal SARS-CoV-2 infection drives alterations in immune cell populations and pro-inflammatory responses at the maternal-fetal interface [73–80] that have the capacity to impact the fetal brain [81]. Even in the absence of direct viral transmission to the fetus, profiling of umbilical cord blood immune cell populations and the serum proteome demonstrates that maternal SARS-CoV-2 infection can shape fetal and neonatal immunity [44, 82–84]. Prior bulk and single-cell transcriptomic analyses have also revealed significant reprogramming at the maternal-fetal interface following SARS-CoV-2 infection during pregnancy [44, 73, 74, 79], yet granular information on fetal placental cell populations has been relatively limited by their lower representation in these studies.

Here we report single-cell RNA-seq data that provide new insights into the heterogeneous functions that fetal placental macrophages, or Hofbauer cells, and maternal resident placental macrophages and monocytes or PAMMs, perform at baseline, and how these programs are altered in the setting of maternal SARS-CoV-2 infection. We found that maternal SARS-CoV-2 infection in pregnancy, even distant from delivery and in the absence of placental infection, was associated with significant alterations in the transcriptional programs of Hofbauer cells. These programs were more significantly impacted than those of maternal placental macrophages, as indicated by number of DEG. Effects of maternal SARS-CoV-2 infection were subcluster-specific, with phagocytosis being a key dysregulated function, and affected Hofbauer cell clusters exhibited signatures consistent with neuroinflammation and neurologic disease. We directly tested this predicted dysregulation using in vitro models of HBC-based induced microglia (HBC-iMGs) [25–27], confirming that SARS-CoV-2 infection altered HBC-iMG function but not other phenotypic characteristics as indicated by preserved morphology. SARS-CoV-2 exposed HBC-iMGs exhibited reduced synaptosome phagocytosis, an assay that serves as a proxy for synaptic pruning. Notably, reduced synaptic pruning by microglia has been identified as a key mechanism in the pathogenesis of a range of neurodevelopmental disorders including autism [61, 85], a neurodevelopmental disease associated with maternal immune activation and viral infection in pregnancy [63, 86, 87]. Thus, the altered resident tissue macrophage programs described here may not be specific to SARS-CoV-2 infection but may reflect more general alterations observed in viral infection in pregnancy.

Although an unavoidable limitation of our study is the inability to directly assay fetal brain microglia of the live offspring from cases and controls, data from mouse models show that HBCs mirror the transcriptional and functional responses of fetal brain microglia to maternal obesity, a chronic inflammatory state [19, 20]. Considering the shared fetal yolk sac origin of HBCs and brain microglia [21, 22], HBCs may serve as a more accessible cell type at birth that could provide information about fetal brain immune programming. The creation of personalized fetal cellular models of neurodevelopment after *in utero* exposure to maternal SARS-CoV-2 infection or other immune-activating exposures has the potential to identify at-risk offspring earlier in life, which in turn could facilitate efforts to normalize neurodevelopmental trajectories [88, 89].

Our study is unique in its in-depth, focused interrogation of fetal immune cell populations of the placenta in the context of a remote maternal viral infection. Through sex-chromosome-specific gene expression mapping, we were able to reliably assign fetal cell identity to 8 subtypes of HBCs, engaged in a myriad of functions at baseline. Similar to prior work in first trimester placenta, we identified subclusters with transcriptional programs associated with angiogenesis and tissue remodeling, as well as clusters enriched for immune defense functions [43]. Concordant with prior single-cell RNA sequencing studies of the placenta in the context of SARS-CoV-2 infection [44, 90], we identified that even in the absence of direct placental infection or active COVID-19 disease at the time of delivery, infection with SARS-CoV-2 in pregnancy, even remote from delivery, had a profound impact on the transcriptional programs of the fetal macrophage population, and to a lesser extent maternal PAMMs.

We defined a broad range of responses to SARS-CoV-2 across HBC subclusters, including some clusters with relatively few DEG and others with significant transcriptional shifts. Impacted HBC subclusters demonstrated transcriptional programs evoking the changes observed in neuroinflammation, and the same subclusters exhibited alterations in phagocytosis and in chemotaxis and cellular movement. To investigate these results further we created induced microglial cellular models from the same samples (HBC-iMG). Phenotypic and functional analyses of HBC-iMGs from SARS-CoV-2 positive samples demonstrated significant impairments in synaptosome phagocytosis. Reduced phagocytic efficiency appeared to result from reduced capacity for synaptosome uptake within the cell, rather differences in the proportion of cells engaged in phagocytosis.

We demonstrated for the first time that HBCs can be used to create microglia-like cell models, applying this approach to gain insight into fetal brain immune programming in the context of maternal SARS-CoV-2 infection. As yolk-sac derived macrophages that colonize the fetal brain early in development [21], microglia play a fundamental role in neurogenesis by promoting neural precursor cell proliferation, axonal outgrowth, and synaptic wiring throughout development [57–59]. A key function of microglia in normal neurodevelopment also includes selective phagocytosis of excess neuronal precursors and synapses to edit and refine the architecture of neuronal communication [59, 91]. Evidence from animal models of maternal immune activation (MIA) suggests that microglia are keenly responsive to maternal innate immune signaling, and MIA-induced disruption of normal microglial function can recapitulate social deficits and other behaviors correlative of those observed in neurodevelopmental disorders such as autism spectrum disorder and schizophrenia [61, 62, 92, 93]. In contrast to induced pluripotent stem cell (iPSC)-based models of fetal microglia [94, 95], which have been shown to disrupt cellular phenotypes and imprinting such that the differentiated cells may not recapitulate the input cell type and consequences of exposure [96, 97], our HBC-based model is derived from the primary tissue itself and does not require long-term passaging, making it more likely to retain epigenetic programming signatures [98]. Patient-specific HBC-iMGs may therefore have advantages over iPSC-derived models for investigating the consequences of maternal exposure on fetal microglia programming. We note, however, that these models are intended to examine microglial behavior in an experimental context, not to precisely recapitulate in vivo processes. In the same way, while phagocytosis assays correlate with pruning in 2D culture [25–27], as a model system they cannot capture the full range of developmental or regional specificity of pruning.

A primary strength of our study is inclusion of rigorously phenotyped individuals without a history of prior SARS-CoV-2 infection or vaccination and of contemporaneously enrolled control subjects. We thus were able to examine the impact of maternal SARS-CoV-2 on an immunologically naïve cohort in the absence of prior immunity to SARS-CoV-2, with a consequence being that we could not assess the impact of prior vaccination. Neither the impact of COVID-19 severity nor fetal sex could be assessed in this study due to the study design (primarily focused on symptomatic infection and male samples as a proof of principle study) and small sample size. Sex differences will be particularly important to assess in future work, given the importance of fetal sex on offspring neurodevelopmental vulnerability and fetoplacental programming [99, 100]. Evaluation of HBC-iMG motility will also be an important future direction to explore given studies linking maternal immune activation to delayed microglial migration [101, 102]. Taken together, our results suggest the ability of HBC-iMGs to serve as personalized cellular models of microglial programming in the setting of maternal exposures, including SARS-CoV-2 and potentially other environmental exposures that might impact neurodevelopment. They demonstrate potential mechanisms by which these exposures may contribute to adverse neurodevelopmental outcomes.

## Methods

### Study design and participant enrollment

In this study, 24 pregnant individuals with full-term, singleton pregnancies delivering at Massachusetts General Hospital (March 2021 - June 2023) were included. Participants were classified as SARS-CoV-2 positive (*N* = 10) if they had symptomatic COVID-19 infection during pregnancy confirmed by positive SARS-CoV-2 nasopharyngeal PCR test, or were asymptomatic but tested positive on routine SARS-CoV-2 screening at delivery. Participants were classified as SARS-CoV-2 negative (*N* = 14) if they did not have a positive SARS-CoV-2 nasopharyngeal PCR or COVID-19 symptoms during pregnancy and had a negative SARS-CoV-2 nasopharyngeal PCR at delivery upon universal COVID-19 screening on Labor and Delivery. Pregnant individuals were eligible for inclusion if they were 18 years or older and were delivering during the COVID-19 pandemic. Exclusion criteria included a history of autoimmune disease or chronic use of immunomodulatory medications. A study questionnaire and review of the electronic health record was used to determine key demographic and clinical variables such as maternal age, gestational age at delivery, gestational age at positive COVID-19 test, COVID-19 disease severity at diagnosis, any prior diagnoses of COVID-19 or history of COVID-19 vaccination, and infant sex and birthweight. Placental pathology was performed for specific clinical indications per institutional protocol [103]. COVID-19 disease severity was assigned as asymptomatic, mild, moderate, or severe using NIH criteria [104], also endorsed for use in pregnancy by the Society for Maternal Fetal Medicine. Manual review of the electronic health records of the offspring in this study was performed to assess for the presence of diagnostic codes associated with abnormal neurodevelopment, as previously described [6, 7].

### Placenta collection and processing

Placentas were obtained within 20 min after delivery and submerged in Cytowash media (Dulbecco’s Modified Eagle Medium (DMEM) containing 2.5% FBS, 1% Penicillin-Streptomycin, 0.1% Gentamicin) and stored at 4 °C until cell isolation. Isolation of Hofbauer cells was performed using previously described protocols [30]; reagents are listed in Supplemental Table S1 and isolation workflow and study procedures are depicted in Supplemental Figure S4. Briefly, placental chorionic villi were separated from fetal membranes and decidua, washed in DPBS wash, and mechanically homogenized. Placental tissue was then serially digested in Collagenase Digestion Buffer, Trypsin Digestion Buffer, and Collagenase Digestion Buffer 2. Undigested tissue was removed by passage through sterile gauze and 100 μm filter. The cell suspension was centrifuged at 257 g for 8 min at 4 °C, washed, spun again, and resuspended in media. The cells were then suspended in 4mL of 20% Percoll and 5mL of 35% Percoll was underlayed through a #1 glass Pasteur pipette [105]. After centrifugation for 30 min at 4 °C without brake at 1000 g, cells were isolated from the interphase layer, washed in media, and spun at 257 g for 8 min at 4 °C. Cell pellets were immunopurified by negative selection by incubation with anti-EGFR (to remove syncytiotrophoblasts) and anti-CD10 (to remove fibroblasts) conjugated to magnetic Dynabeads, prepared as previously described (76), for 20 min at 4 °C. Tubes were placed on a DynaMagTM magnet for 5 min to magnetically bind cytotrophoblasts (anti-EGFR) and fibroblasts (anti-CD10) – allowing media containing unbound placental macrophages to pass through into collection tubes. Cells were cryopreserved in 90% FBS and 10% dimethyl sulfoxide (DMSO) at 1–10 million cells/vial and stored at -80 °C for downstream analyses. SARS-CoV-2 viral loads were assessed in all placental tissues using qPCR as previously described, with 40 copies/mL as limit of detection [14, 28].

### Single-cell RNA-sequencing (scRNA-Seq) data analysis

*Sequencing.* Cryopreserved HBCs were thawed at 37 °C and diluted with RPMI 1640 including 10% FBS and 1% Pen/Strep. The cell suspension was centrifuged at 300 g for 5 min at room temperature, with the brake off. The supernatant was aspirated and the cell pellet was resuspended in media. Dead cells were removed using OptiPrepTM Density Gradient Medium (Sigma) and cell count and viability of cells were calculated using LunaFX7 automated counter. Cells were immediately loaded onto 10x Genomics platform with a loading target of approximately 10,000 viable cells/sample. Libraries were sequenced on an Illumina NextSeq 2000 P3 flowcell machine with a sequencing target of 25,000 reads per cell.

*Initial cluster identification*. Raw reads were aligned to reference genome GRCh38 and quantified using Cell Ranger (version 6.0.1, 10x Genomics) and after initial cellranger filtering an average of 6,295 cell/sample and 20,459 reads/cell were present. Putative doublet cells were removed using predictions generated from DoubletFinder (v2.0.3) as were cells containing less than 300 identified genes, which resulted in an object containing 70,817 cells. All samples were integrated to remove batch effects from individuals using the Seurat Single Cell Transform workflow (Seurat version 4.3.0) with the top 2,000 variable features. Cells were clustered using the Louvain algorithm on the shared nearest neighbor graph and visualized by UMAP using the first 30 principal components. Several clustering resolutions were used to scan through the data to identify a resolution (0.3) that allowed us to identify top-level cell types based on marker genes. Marker genes were identified using the Wilcoxon rank sum test with the following parameters: only.pos = TRUE, min.pct = 0.2, logfc.threshold = 0.5. Additionally, expression of well-known cell-type markers was assessed to refine top-level identities: hofbauer, fibroblasts, NK cells/CD8 T cells, VECs (vascular endothelial cells), EVTs (extravillous trophoblasts), RBCs (red blood cells), B cells, and neutrophils (Supplemental Figure S1). For subsequent analyses, we created a subset of the data including only cells identified as “Hofbauer,” which included macrophage and monocyte populations.

*Subcluster analysis.* The data were re-integrated and processed similarly as described above to identify macrophage/monocyte subclusters. Only high-quality cells were retained (mitoRatio < 0.25 and nUMI > 1000 and < 9681, or 3 standard deviations above the mean UMI count). The number of subclusters was optimized by iteratively clustering across several cluster resolutions, and identifying the resolution that provided non-redundant clusters (resolution = 0.3) as determined by marker gene identification with Seurat’s Wilconcon rank-sum test (only.pos = TRUE, min.pct = 0.3, and logfc.threshold = 0.5). Subclusters were then assigned as HBCs (0–7), PAMMs or Monocytes based on marker genes. To delineate fetal from maternal origin of subclusters, we evaluated sex-specific markers using only cells from placentas with a male fetus (*N* = 10). This allowed for maternal vs. fetal cell differentiation, as fetal cells would be expected to have increased expression of the male-specific Y-linked gene *DEAD-Box Helicase 3 Y-Linked* (*DDX3Y*) and maternal cells would exhibit high expression of the female-specific gene *X-inactive specific transcript* (*XIST*). The macrophage cluster with high expression of *XIST* consistent with maternal origin was annotated as maternal placenta-associated macrophages and monocytes (PAMMs). To further support cell cluster annotation, we also compared the overall gene expression profiles of each cluster to a previously published single-cell dataset derived from human first-trimester placenta and decidua [32].

*Differential gene expression by SARS-CoV-2 status*. For differential gene expression analysis between cells from SARS-CoV-2 positive cases and negative controls, we used the Seurat FindMarkers() within each cluster with the following parameters: test.use = “MAST”, min.pct = 0.3, logfc.threshold = 0.2, latent.vars = “donor”. Genes with Benjamini-Hochberg adjusted p-value < 0.05 were considered significant. Gene lists used to compute module scores in Fig. 3E for comparison to Bian et al. [64] and Askenase et al. [65] datasets are given in Supporting Data Values File (S7).

*Functional enrichment analyses.* Gene Ontology (GO) Biological Process enrichment analysis was performed on both cluster marker genes and SARS-CoV-2 differentially expressed genes in each cluster using R package clusterProfler (v. 3.18.1) [106] and underlying database AnnotationDb org.Hs.eg.db (v3.12.0). GO terms were considered significant with adjusted p-value < 0.05. IPA Canonical Pathway and Diseases and Functions analysis were performed with IPA (Content Version: 90,348,151) with pathways considered significant with adjusted p-value < 0.05.

### Derivation of Hofbauer cells transdifferentiated toward microglia-like cells (HBC-iMGs) by direct cytokine reprogramming

HBC-iMGs were derived from HBCs using previously described methods [25–27], with modifications as noted. Briefly, thawed HBCs were plated on Geltrex-coated 24-well plates (1 × 10^6^ cells in 0.5 mL per well) or 96-well plates (2 × 10^5^ cells in 0.1mL per well) depending on cell availability. After cells were incubated at 37 °C for 24 h, the media was completely replaced with RPMI 1640 including GlutaMAX, 1% penicillin–streptomycin, 100 ng/mL of human recombinant IL-34 (Peprotech), and 10 ng/mL of GM-CSF (Peprotech). At day 6 of transdifferentiation, the cultures were assayed and subsequently fixed with 4% PFA to perform endpoint analysis using immunocytochemistry.

### HBC-iMG immunocytochemistry

HBC-iMGs were washed twice with PBS and blocked for 1 h with 5% FBS and 0.3% Triton-X (Sigma Aldrich) in PBS at room temperature. Next, they were washed three times with 1% FBS in PBS and incubated with primary antibodies in 5% FBS and 0.1% Triton-X overnight at 4 °C (Anti-IBA1, 1:500; Abcam #ab5076; Anti-TMEM119, 1:500; Abcam #ab18537; Anti-CX3CR1, 1:100, Abcam #ab8021; Anti-PU.1, 1:1000, Abcam #ab183327, and Anti-P2RY12, 1:100, Alomone Labs). Cells were then washed three times with 1% FBS in PBS and incubated in secondary antibodies (Invitrogen Alexa Fluor, 1:500) and Hoechst 33,342 (1:5000) in 5% FBS and 0.1% Triton-X in PBS for 45 min at 4 °C. Cells were washed two final times and imaged using the IN Cell Analyzer 6000 (Cytiva). Proportion of labeled cells was analyzed using CellProfiler [107] as described below. Cells were segmented using one of the four microglia markers used and percent marker positive cells calculated by dividing the number of marker positive cells by the number of identified nuclei, per image. A total of 12 20x images per sample were analyzed.

### Synaptosome derivation and phagocytosis assay

*Synaptosome generation from neural progenitor cell cultures.* Induced pluripotent stem cells were reprogrammed from fibroblasts and used to derive expandable neural progenitor cells, and large-scale differentiated neural cultures, as previously described [25–27]. After media removal, neural cultures were collected by scraping in 10 ml per T1000 flask 1× gradient buffer (ice-cold 0.32 M sucrose, 600 mg/L Tris, 1 mM NaH3CO3, 1 mM EDTA, pH 7.4 with added HALT protease inhibitor—Thermo-Fisher Scientific # 78,442) and homogenized using a Dounce Tissue Grinder (Wheaton #357,544 15 ml) with the ‘tight’ plunger. Homogenate was collected and centrifuged at 700 g g for 10 min at 4 °C to remove large debris. The gradient buffer was removed by aspiration and saved on ice then the pellet was resuspended in 10 mL of 1× gradient buffer and homogenization was repeated as above. The final homogenates were combined and centrifuged at 15,000 g for 15 min at 4 °C. This second pellet was resuspended in 12 ml 1× gradient buffer and slowly added on top of a pre-formed sucrose gradient in Ultracentrifuge Tubes (Beckman Coulter Ultra-Clear #344,058) containing 12 ml each 1.2 M (bottom) and 0.85 M (middle) sucrose layers. The gradients were centrifuged using Ultracentrifuge swinging bucket rotor #SW32TI at 26,500 RPM (~ 80,000 g) for 2 h at 4 °C with the brake off. The synaptosome band (in between 0.85 and 1.2 M sucrose layers) was removed using a 5-mL syringe and 19Gx1 ½″ needle, diluted with 5-fold 1x gradient buffer then centrifuged at 20,000 g for 20 min at 4 °C. The final pellet was resuspended in an appropriate volume of 1× gradient buffer containing 1 mg/mL bovine serum albumin (BSA) with HALT protease and phosphatase inhibitors added, aliquoted and slowly frozen at − 80 °C. Protein concentration was measured by BCA and enrichment of pre-synaptic (synapsin, SNAP-25) and post-synaptic (PSD-95) markers was determined by western blot analysis.

*Phagocytosis Assay.* Synaptosomes were thawed and labeled with pHrodo Red SE (Thermo-Fisher Scientific #P36600) at 1:2 (mg dye: mg synaptosome) and incubated at room temperature for 1 h. Labeled synaptosomes were centrifuged at 15,000 g for 15 min to wash away unbound pHrodo Red SE. They were resuspended in RPMI 1640 and then sonicated for 1 h before adding to HBC-iMGs at 15 mg total protein per well in 24-well plates, or 3 mg per well in 96-well format. HBC-iMGs with synaptosomes were incubated at 37 °C for three hours and then fixed with 4% PFA for 15 min at room temperature. Immunocytochemistry was performed to quantify phagocytosis, with images analyzed in CellProfiler (v4.2.4). HBC-iMGs were segmented as described below using IBA1 staining and phagocytic index was calculated by dividing the signal area of pHrodo Red by the number of segmented cells, per image.

### Image analysis

CellProfiler (v4.2.4) was used to identify cellular and subcellular structures in the confocal images [107]. The module CorrectIlluminationCalculate and CorrectIlluminationApply were used in all channels to correct uneven illumination and uneven background. Nuclei and cell bodies were each identified using IdentifyPrimaryObjects. Specifically, pixel diameter ranges and the automatic thresholding method Otsu were applied. The module RelateObjects was used to drop structures incorrectly identified as nuclei by ensuring they were only accepted when they have a surrounding microglia-like cell. IdentifySecondaryObjects was used to more accurately outline cells around these nuclei and avoid debris. The module IdentifyTertiaryObjects identified cytoplasm by subtracting the area of the nucleus from the cell. IdentifySecondaryObjects was used with Otsu thresholding to identify and measure synaptosomes. Background red signal was eliminated by increasing the lower bounds on the automatic threshold, using reference images from Cytochalasin treatment as a positive control of diminished phagocytosis. MaskObjects was used with the cell and synaptosome objects to omit red signal from outside the cell. Overlays of the outlines of all generated image structures were created for quality check purposes using the OverlayOutlines module. Cell area, count, and signal intensity were created with MeasureObjectSizeShape, MeasureObjectIntensity, ExportToSpreadSheet, and ExportToDatabase. RStudio 2 (1.4) was used to organize the metadata exported from CellProfiler. Phagocytic index was calculated as area of Synaptosomes divided by cell count per image. As a confirmation, the integrated intensity of Synaptosome signal divided by cell area was also checked to make sure both measures corresponded. Images containing > 80 cells were omitted due to procedure inaccuracy with dense fields. Outliers were excluded by calculating a phagocytic index threshold of 3 SD above the mean. Morphology data was produced using cell level metadata from CellProfiler followed by a cleaning process matching the field level dataset cleaning. Cells with an area or synaptosome area of greater than the mean plus 3 SD were omitted. In total, at least 12 20x images per sample were analyzed.

### Statistics

Group differences were assessed using Mann-Whitney U tests, with the exception of cellular morphology, which was analyzed at the level of single cells, and phagocytosis, analyzed at the level of individual fields, using linear mixed effects models (via the *lme4* package in R). Nominal two-tailed P values less than 0.05 were considered statistically significant. Dark lines represent median and dotted lines interquartile range, unless otherwise specified. Statistical analyses were performed in GraphPad Prism (version 9.3) and R (version 4.2.3).

### Electronic supplementary material

Below is the link to the electronic supplementary material.

Supplementary Material 1

Supplementary Material 2

## Data Availability

Sequencing data will be made available for download on GEO upon acceptance. Supporting Data Values are available as a supplemental .XLSX file. R code supporting the conclusions of this manuscript is made available here: https://github.com/rbatorsky/covid-placenta-edlow.
