# Supplementary material for: Maternal SARS-CoV-2 impacts fetal placental macrophage programs and placenta-derived microglial models of neurodevelopment
Source: J Neuroinflammation. 2024 Jun 25;21:163. doi: 10.1186/s12974-024-03157-w (PMC11197235; doi:10.1186/s12974-024-03157-w)
Supplement: Supplementary file 1 — Supplementary Material 1 [file 12974_2024_3157_MOESM1_ESM.docx]

**SUPPLEMENTAL INFORMATION**

**Figure S1.**


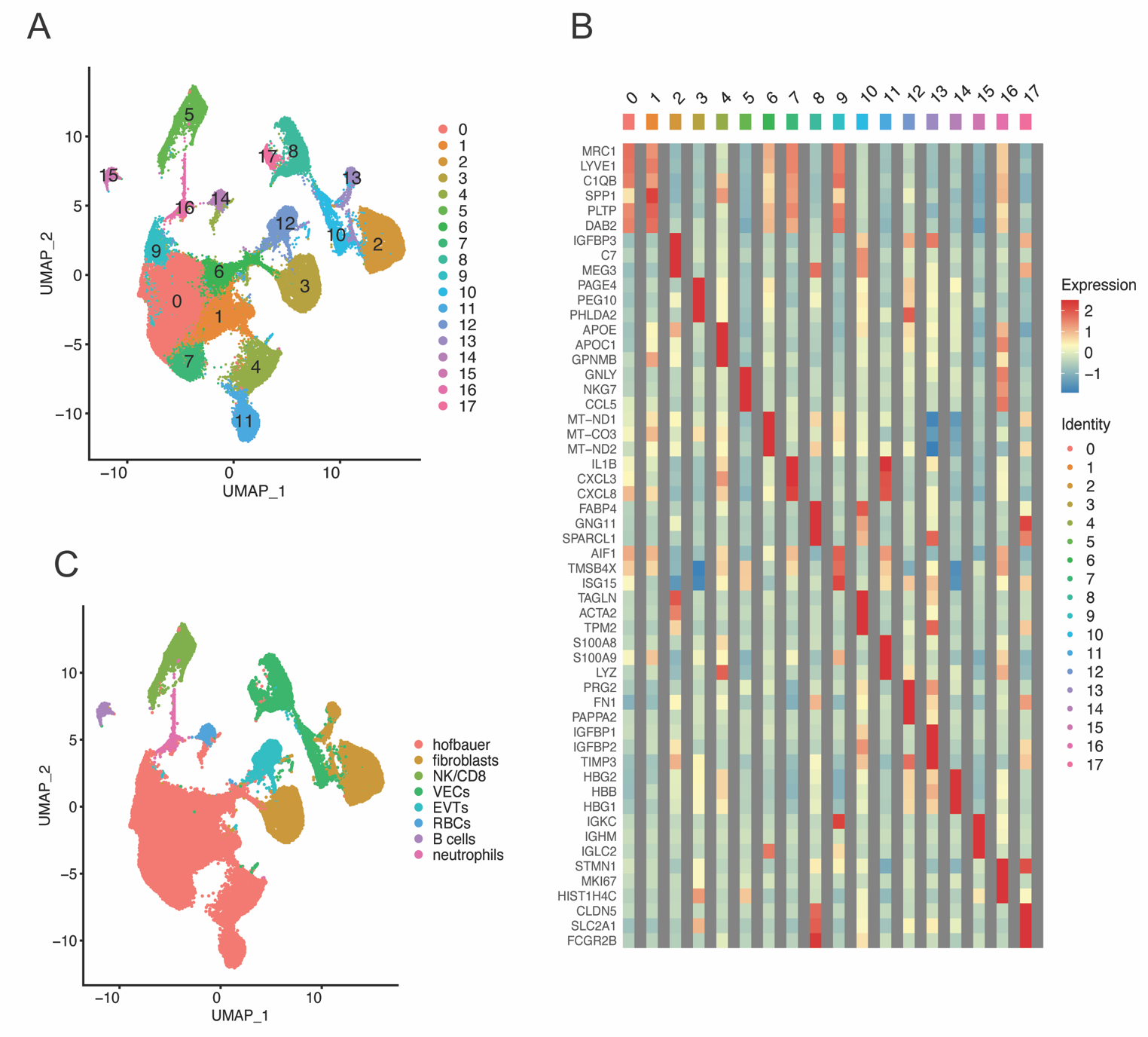


**Figure S1. Initial graph-based clustering and cluster assignments.** (**A**) Uniform Manifold Approximation and Projection (UMAP) visualization of 70,817 high-quality placental cells, enriched from placentas of pregnancies with (n=4) and without (n=8) SARS-CoV-2 infection, shows 18 clusters. (**B**) Heatmap displaying expression (log_2_ fold change) of the top 3 marker genes per cluster. (**C**) UMAP visualization of cluster assignment by marker gene analysis demonstrating largest proportion of cells were macrophages/monocytes (labeled “hofbauer”) and subset for further quality control and reclustering. NK: natural killer cells. VECs: vascular endothelial cells. EVTs: extravillous trophoblasts. RBCs: red blood cells.

**Figure S2.**

**
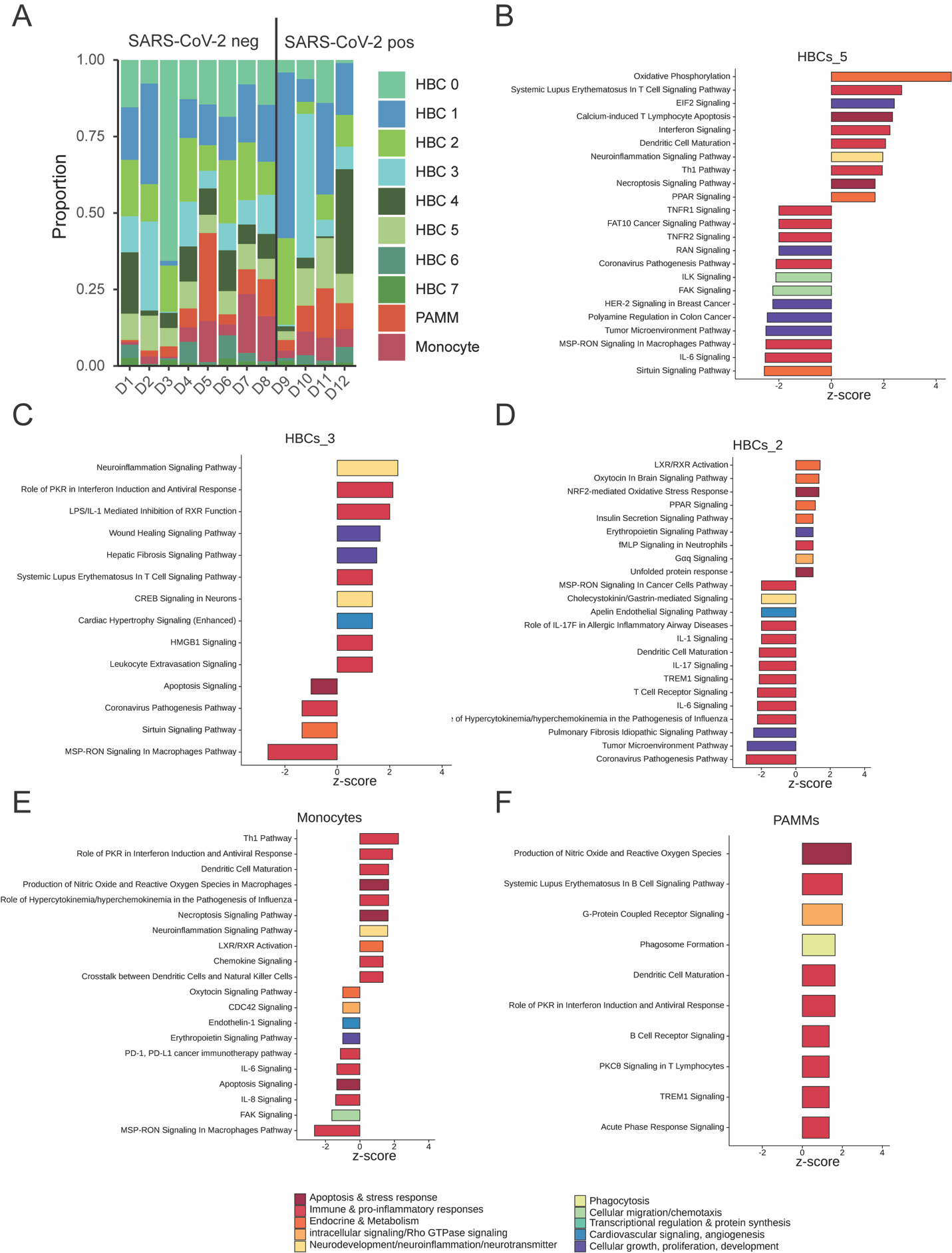
**

**Figure S2.** **Impact of maternal SARS-CoV-2 infection on Hofbauer cell subclusters, PAMMs and monocytes.** HBC: Hofbauer cell. PAMM: placenta-associated macrophage/monocyte. (**A**) Barplot demonstrating proportion of cells within each subcluster by participant. “SARS-CoV-2 neg” = no maternal SARS-CoV-2 infection during pregnancy. “SARS-CoV-2 pos” = positive maternal SARS-CoV-2 test during pregnancy. (**B-F**) Ingenuity Pathway Analysis (IPA) canonical pathways enrichment results of DEG by subcluster. IPA canonical pathways with absolute Z-score ≥1 and adjusted P-value < 0.05 are displayed.

**Figure S3.
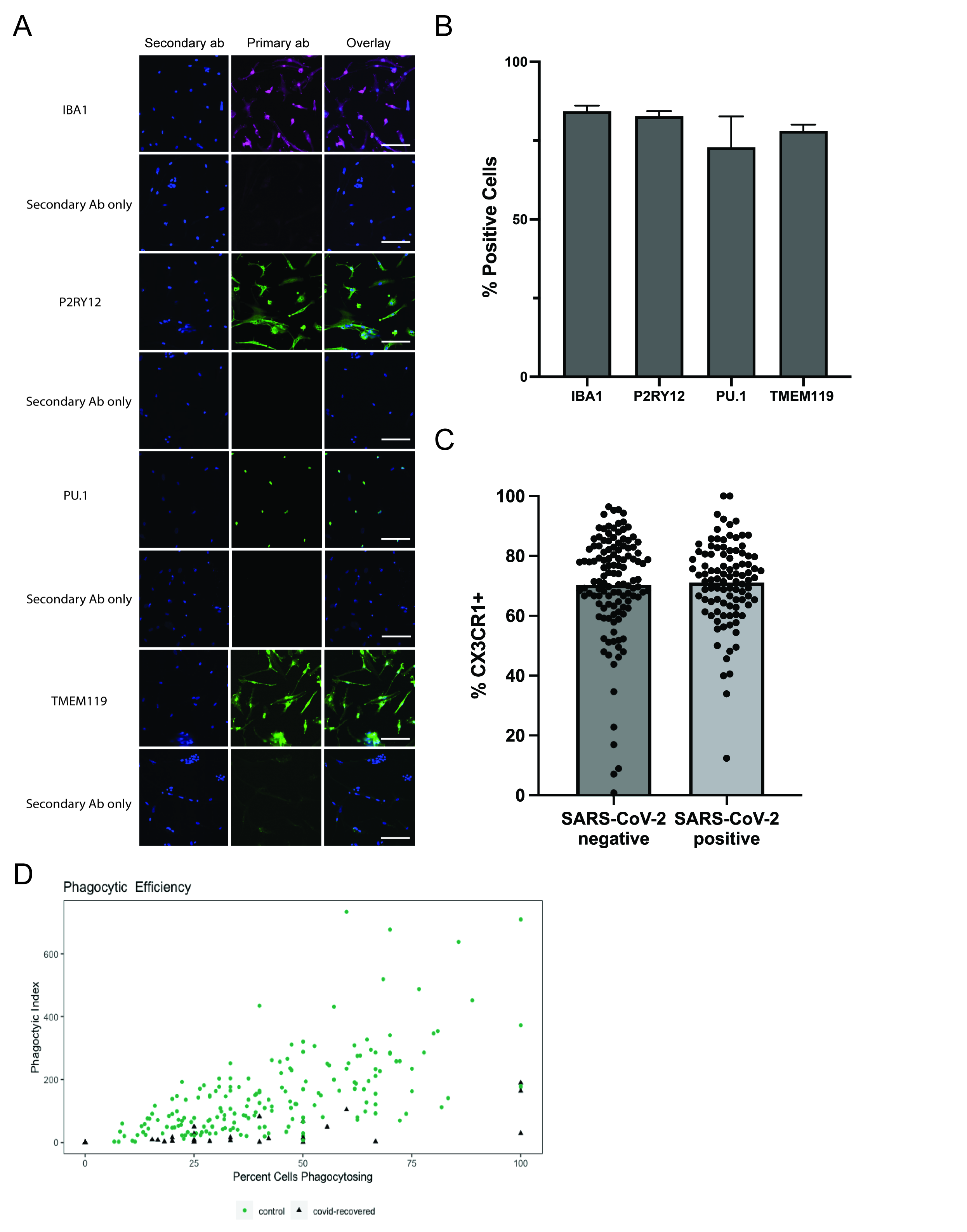
**

**Figure S3. HBC-iMG phenotypic and functional characterization.** HBC-iMGs: Hofbauer cells transdifferentiated toward microglia-like cells. Ab = antibody. (**A**) Confocal microscopy of immunostaining with primary antibody, secondary antibody (Hoechst nuclear stain) and overlay, showing lack of nonspecific staining. Scale bar = 100 µm. (**B**) Percentage of cells per sample with positive immunostaining. (**C**) Percentage of cells per image with positive immunostaining for CX3CR1, by SARS-CoV-2 status (n=98 cases and 122 control images, n= 4 case and 5 control samples). (**D**) Phagocytic Index by percent of cells engaged in any phagocytosis, by SARS-CoV-2 status. Green circles: SARS-CoV-2 negative controls (n=187 fields). Black triangles: SARS-CoV-2 positive cases (n=32 fields).

**Figure S4.**

**
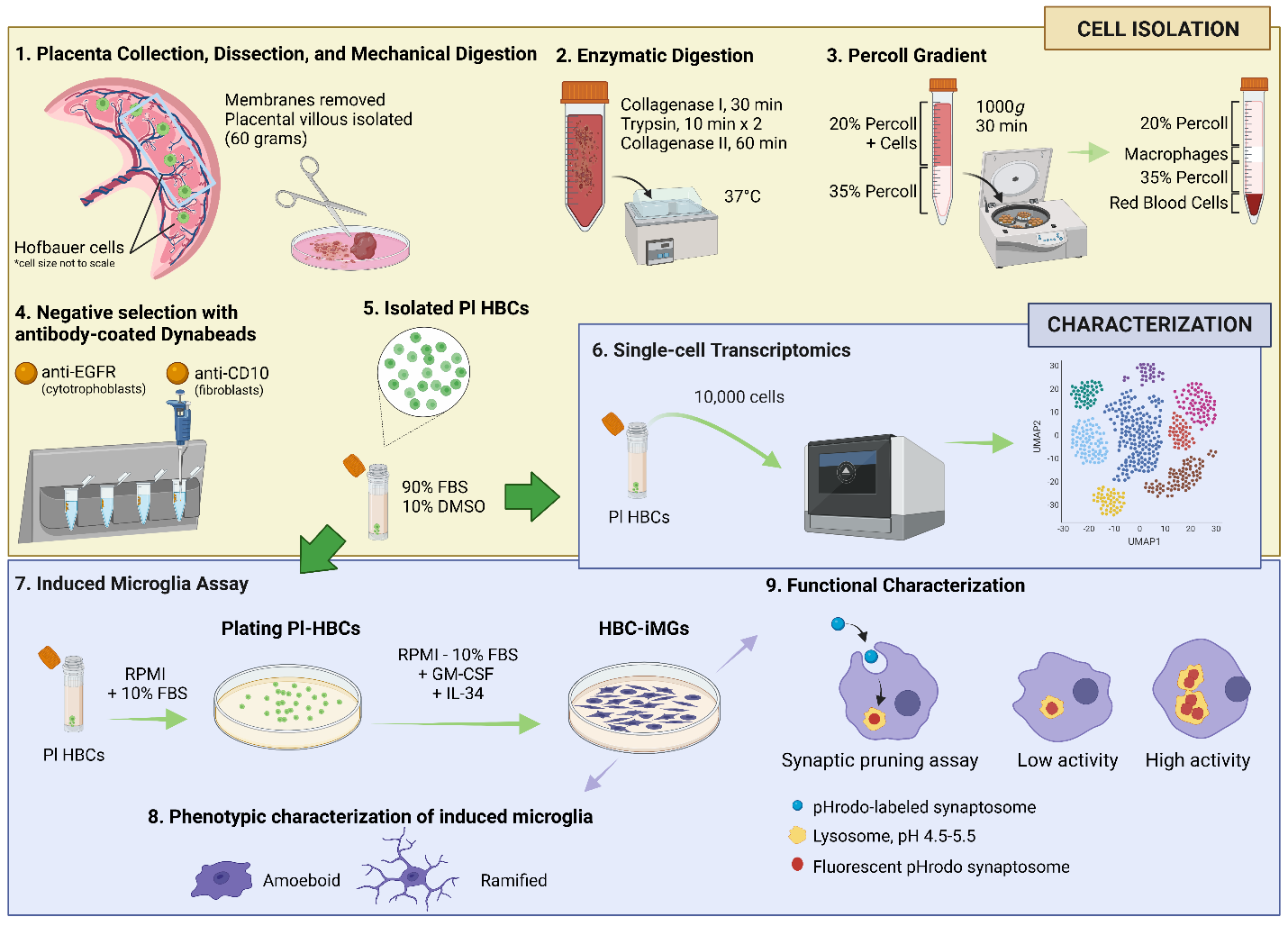
**

**Figure S4. Schematic of Hofbauer cell isolation procedure workflow and downstream analyses.** HBCs: Hofbauer cells. HBC-iMGs: Hofbauer cells transdifferentiated toward microglia-like cells. HBCs were isolated using collagenase and trypsin digestions, followed by centrifugation on Percoll gradients, and negative bead-based selection with antibody-coated anti-EGFR and anti-CD10 Dynabeads. HBCs were then characterized by single-cell RNA-Seq (10X Genomics) and cytokine induction to create HBC-iMGs. Image created in BioRender.

**Table S1. List of reagents used in Hofbauer cell isolation procedure.**

| **Reagents** | **Source** | **Cat#** |
| --- | --- | --- |
| **Cytowash**  DMEM Medium  Fetal Bovine Serum  Pen/Strep (10,000 U/mL)  Gentamicin | Thermo Fisher Scientific  Cytiva  Thermo Fisher Scientific  Thermo Fisher Scientific | 11965118  SH 30071.03  15140122  15750060 |
| **DPBS Wash**  1X DPBS, Ca-/Mg-free  Pen/Strep (10,000 U/mL) | Thermo Fisher Scientific  Thermo Fisher Scientific | 14190144  15140122 |
| **Collagenase Digestion 1**  1X DPBS, Ca-/Mg-free  Collagenase Type IA  DNase I  Hyaluronidase  Bovine Serum Albumin  CaCl (3M stock, 1000x) | Thermo Fisher Scientific  Millipore Sigma  Millipore Sigma  Millipore Sigma  Millipore Sigma  Boston Bio Products | 14190144  11088793001  DN25-1G  H3506-1G  A7906  MT-140-3M |
| **Trypsin Digestion**  1X DPBS, Ca-/Mg-free  Trypsin  DNase I  CaCl (3M stock, 1000x) | Thermo Fisher Scientific  Thermo Fisher Scientific  Sigma Aldrich  Boston Bio Products | 14190144  Gibco 27250018  DN25-1G  MT-140-3M |
| **Collagenase Digestion 2**  1X DPBS, Ca-/Mg-free  Collagenase Type IA  DNase I  Hyaluronidase  Bovine Serum Albumin  CaCl (3M stock, 1000x)  Dispase II | Thermo Fisher Scientific  Millipore Sigma  Millipore Sigma  Millipore Sigma  Millipore Sigma  Boston Bio Products  Thermo Fisher Scientific | 14190144  11088793001  DN25-1G  H3506-1G  A7906  MT-140-3M  171105041 |
| **Gradient Solutions**  Percoll  Hanks’ Balanced Salt Solution 10X  Hanks’ Balanced Salt Solution 1X  OptiPrep Density Gradient Medium | GE Healthcare Biosciences  Thermo Fisher Scientific  Thermo Fisher Scientific  Millipore Sigma | 17-0891-01  14185052  14175095  D1556-250mL |
| **Dynabeads**  Goat anti-mouse IgG antibody conjugated Dynabeads  Mouse anti-human EGFR  Mouse anti-human CD10 antibody | Thermo Fisher Scientific  Thermo Fisher Scientific  Thermo Fisher Scientific | 11033  MA5-16944  MA5-13070 |

**Table S2. Neurodevelopmental outcomes of children.**

| **Group** | **Diagnosis, ICD-10 Diagnostic Code*** |
| --- | --- |
| SARS-CoV-2 positive  (N=10) | Autism spectrum, F84.0 (n=1)  Developmental Delay, R62.50 (n=1) |
| SARS-CoV-2 negative  (N=14) | Expressive Speech Delay, F80.1 (n=1)  Gross Motor Development Delay, F82 (n=1)  Developmental Delay, R62.50 (n=2)  Abnormal Neurological Exam, R29.90 (n=1) |

“Group” refers to maternal infection status during pregnancy. *ICD-10 Diagnostic codes relevant to child neurodevelopment identified in the electronic health records of offspring as previously described (Edlow et al., “Neurodevelopmental Outcomes at 1 Year in Infants of Mothers Who Tested Positive for SARS-CoV-2 During Pregnancy,” *JAMA Network Open* 5, no. 6 (June 1, 2022): e2215787.). 3 years of follow-up data were available for 22 of 24 infants; 2 infants had follow-up at 10 and 25 months of life. No child had more than one diagnostic code assigned.

**SUPPORTING DATA VALUES: SHEET INFORMATION.**

**File S1: Complete list of marker genes for subset clusters.**

Column headings:

p_val : unadjusted p-value

avg_log2FC : log fold-change of the average expression of the gene between all cells in the cluster being considered and all cells in other clusters

pct.1 : The percentage of cells where the gene is detected in the cluster being considered

pct.2 : The percentage of cells where the gene is detected in all other clusters

p_val_adj : Adjusted p-value by the Benjamini-Hochberg method

**File S2: Complete list of GO biological pathway enrichment results for subset clusters.**

Column headings:

GeneRatio: ratio of the number of input genes that are annotated with the GO term with the total number of input genes

BgRatio: ratio of the number of genes that are annotated with the GO term with all genes in the database annotated with the GO term

p.adjust: Adjusted p-value by the Benjamini-Hochberg method

geneID: List of input genes that are annotated in a term

Count: number of input genes that are annotated in a term

**File S3. Complete list of DEG by SARS-CoV-2 for subset clusters.**

Column headings:

p_val : unadjusted p-value

avg_log2FC : log fold-change of the average expression of the gene between all cells in the cluster being considered and all cells in other clusters

pct.1 : The percentage of cells where the gene is detected in the cluster being considered

pct.2 : The percentage of cells where the gene is detected in all other clusters

p_val_adj : Adjusted p-value by the Benjamini-Hochberg method

**File S4. Complete list of GO biological pathways enrichment results for DEG by SARS-CoV-2 for subset clusters.**

Column headings:

GeneRatio: ratio of the number of input genes that are annotated with the GO term with the total number of input genes

BgRatio: ratio of the number of genes that are annotated with the GO term with all genes in the database annotated with the GO term

p.adjust: Adjusted p-value by the Benjamini-Hochberg method

geneID: List of input genes that are annotated in a term

Count: number of input genes that are annotated in a term

**File S5. Complete list of IPA canonical pathways enrichment results for DEG.**

Column headings:

z-score: indicates a predicted activation or inhibition of a pathway/gene, where a negative z value connotates an overall pathway's inhibition, and a positive z value connotates an overall pathway's activation

Molecules: list of input genes in the pathway

**File S6. Complete list of IPA diseases and functions enrichment results for DEG.**

Column headings:

z-score: indicates a predicted activation or inhibition of a pathway/gene, where a negative z value connotates an overall pathway's inhibition, and a positive z value connotates an overall pathway's activation

Molecules: list of input genes in the pathway

**File S7. Complete list of genes in each module depicted in Fig. 3D.**

**File S8. Additional participant clinical information and placental pathology.**
